# Large‐Scale Production of Wholly Cellular Bioinks via the Optimization of Human Induced Pluripotent Stem Cell Aggregate Culture in Automated Bioreactors

**DOI:** 10.1002/adhm.202201138

**Published:** 2022-11-22

**Authors:** Debbie L. L. Ho, Stacey Lee, Jianyi Du, Jonathan D. Weiss, Tony Tam, Soham Sinha, Danielle Klinger, Sean Devine, Art Hamfeldt, Hope T. Leng, Jessica E. Herrmann, Mengdi He, Lee G. Fradkin, Tze Kai Tan, David Standish, Peter Tomasello, Donald Traul, Noushin Dianat, Rukmini Ladi, Quentin Vicard, Kishore Katikireddy, Mark A. Skylar‐Scott

**Affiliations:** ^1^ Department of Bioengineering Stanford University Stanford CA 94305 USA; ^2^ Sartorius Stedim North America Inc 565 Johnson Avenue Bohemia NY 11716 USA; ^3^ School of Medicine Stanford University Stanford CA 94305 USA; ^4^ Materials Science and Engineering Stanford University Stanford CA 94305 USA; ^5^ Institute of Stem Cell Biology and Regenerative Medicine Stanford University School of Medicine Stanford CA 94305 USA; ^6^ Department of Genetics Stanford University School of Medicine Stanford CA 94305 USA; ^7^ Department of Pathology Stanford University School of Medicine Stanford CA 94305 USA; ^8^ Sartorius Stedim France S.A.S Zone Industrielle les Paluds Avenue de Jouques CS 71058 Aubagne Cedex 13781 France; ^9^ Basic Science and Engineering Initiative Children's Heart Center Stanford University Stanford CA 94305 USA; ^10^ Chan Zuckerberg Biohub San Francisco CA 94158 USA

**Keywords:** 3D bioprinting, cell manufacturing, organoids, pluripotent stem cells, suspension culture

## Abstract

Combining the sustainable culture of billions of human cells and the bioprinting of wholly cellular bioinks offers a pathway toward organ‐scale tissue engineering. Traditional 2D culture methods are not inherently scalable due to cost, space, and handling constraints. Here, the suspension culture of human induced pluripotent stem cell‐derived aggregates (hAs) is optimized using an automated 250 mL stirred tank bioreactor system. Cell yield, aggregate morphology, and pluripotency marker expression are maintained over three serial passages in two distinct cell lines. Furthermore, it is demonstrated that the same optimized parameters can be scaled to an automated 1 L stirred tank bioreactor system. This 4‐day culture results in a 16.6‐ to 20.4‐fold expansion of cells, generating approximately 4 billion cells per vessel, while maintaining >94% expression of pluripotency markers. The pluripotent aggregates can be subsequently differentiated into derivatives of the three germ layers, including cardiac aggregates, and vascular, cortical and intestinal organoids. Finally, the aggregates are compacted into a wholly cellular bioink for rheological characterization and 3D bioprinting. The printed hAs are subsequently differentiated into neuronal and vascular tissue. This work demonstrates an optimized suspension culture‐to‐3D bioprinting pipeline that enables a sustainable approach to billion cell‐scale organ engineering.

## Graphical abstract

Practicing the art of manufacturing densely cellular solid organs on demand will require the production of billions of stem cells. Here, an optimized pipeline is presented for growing up to 4 billion human induced pluripotent stem cells in automated bioreactor systems. These stem cell aggregates are compacted into viscoelastic and wholly cellular bioinks and are subsequently bioprinted and differentiated.

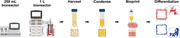

## 1 Introduction

The promise of biomanufactured autologous tissues and organs on demand has been a long‐standing challenge in the field of tissue engineering. The absence of readily perfusable vascular networks had limited the scale at which traditional scaffold‐based approaches could generate viable solid organ tissues.^[^ ^1^ ^]^ However, recent and rapid progress in 3D bioprinting is not only resulting in complex 3D, anatomically accurate components, but critically, it can integrate pervasive, branched, and perfusable vascular networks into tissues, essentially unlocking the third dimension of tissue biofabrication.^[^ ^2^, ^3^, ^4^, ^5^, ^6^, ^7^ ^]^ However, while bioprinting technologies are now poised to take bold steps toward organ‐scale manufacturing, bioengineers are now critically limited by a lack of cellular material^[^ ^8^ ^]^ and the need to render bioinks that can recapitulate the cellular density and organization of solid organ tissues. Specifically, it is estimated that human adult‐sized solid organs contain 10–300 billion cells and have cellular densities of approximately 100 million cells per mL.^[^ ^2^, ^5^, ^9^ ^]^ For example, the human heart is estimated to contain 2–3 billion cardiac muscle cells,^[^ ^10^, ^11^ ^]^ and the human liver is estimated to consist of 160–360 billion cells.^[^ ^12^, ^13^ ^]^ The density of compacted human induced pluripotent stem cell aggregate (hA) slurries is approximately 200 million cells mL^−1^, which is close to native tissues such as liver (150 million hepatocytes mL^−1^, not including other stromal cells^[^ ^14^ ^]^) and heart (100–500 million cells mL^−1[^ ^15^ ^]^). Furthermore, a successful organ engineering process will inevitably require the printing of not just one, but thousands of organs to hone the printing and perfusion parameters, incorporated cell types, extracellular matrix composition, maturation media, and quality control procedures, among countless other variables. Thus, we posit that the development of a scalable cell manufactory‐to‐bioprinter pipeline will be an essential driver of efficient progress in organ engineering.

Human induced pluripotent stem cells (hiPSCs) offer tremendous potential for 3D bioprinting tissues because of their capacity for generating limitless autologous differentiated cell types.^[^ ^16^, ^17^, ^18^, ^19^ ^]^ Furthermore, their ability to spontaneously form 3D hAs that can proliferate, undergo serial passaging, and differentiate into self‐assembled organoids entirely in suspension culture, offers a truly scalable process for engineering organs replete with organ‐like microarchitecture.^[^ ^9^ ^]^ Automated stirred tank bioreactors are the industry standard for scalable bioprocessing, and have been developed for the large‐scale production of microbial and mammalian cells, and more recently, embryonic stem cells and hiPSCs.^[^ ^20^, ^21^, ^22^ ^]^ Bioreactors offer real‐time, traceable monitoring and closed‐loop control of growth parameters including agitation, temperature, pH, dissolved oxygen (DO), and media feed rates, which are critical for maintaining optimal growth conditions and are required to adhere to the Food and Drug Administration's Current Good Manufacturing Practices (cGMP) guidelines.^[^ ^23^, ^24^ ^]^ These growth parameters can be tuned to tailor hA sizes according to downstream application requirements, such as subculturing or differentiation, while ensuring that pluripotency is maintained throughout the hA.^[^ ^25^, ^26^, ^27^ ^]^ Thus, automated bioreactors could serve as ideal inputs to clinical‐grade and organ‐scale bioprinting pipelines.

Once hAs are derived at scale, their compact and densely cellular nature allows them to be rendered into wholly cellular bioinks. By centrifuging hAs, the resulting pellet behaves like a jammed granular material with the requisite yield stress, viscoelastic, and shear thinning rheology for extrusion 3D bioprinting.^[^ ^5^ ^]^ Of note, the rheology is dominated by the jammed nature of the hAs themselves, and does not require the addition of xenogenic or synthetic biomaterials to achieve a printable formulation. This wholly cellular bioprinting method could offer significant advantages in avoiding the high cost associated with purified or recombinant extracellular matrix materials and for evading safety risks and deleterious immune responses post‐transplantation that are associated with xenogeneic and allogeneic materials.^[^ ^28^ ^]^ However, a detailed study of the rheology of densely packed singular hiPSCs or bioreactor‐derived hAs, and the ability to bioprint and differentiate the wholly cellular hA bioink into multiple lineages has not been conducted.

**Figure 1:**
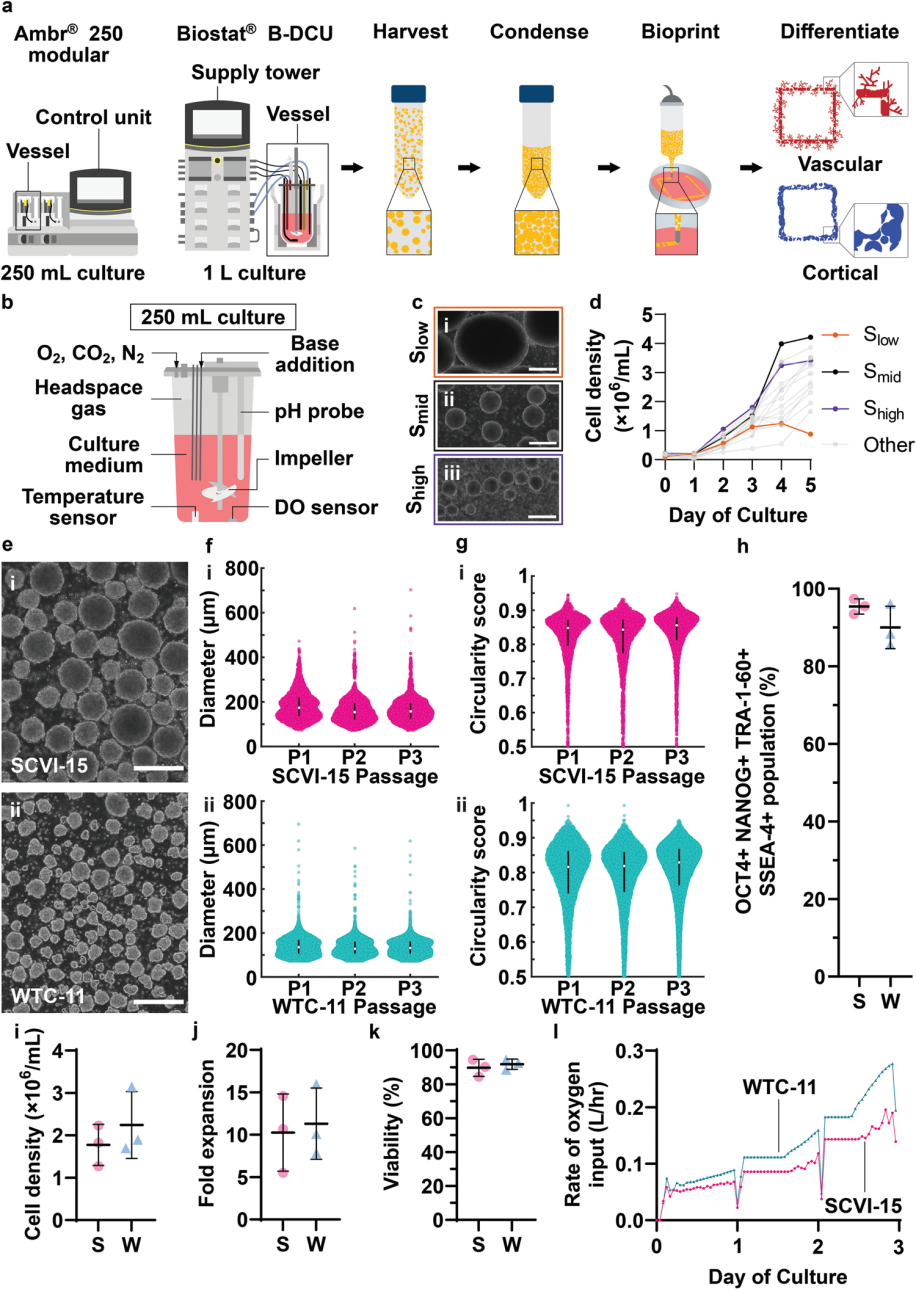
Optimizing bioreactor culture parameters of hiPSC aggregates for fueling bioprinting pipelines. a) Schematic of 250 mL and 1 L automated suspension culture bioreactors and workflow to produce hAs for wholly‐cellular bioprinting and differentiation. On the day of harvest, hAs are collected, centrifuged to form a bioink slurry, loaded into a syringe and 3D bioprinted through a nozzle, and differentiated into organ‐specific tissues. b) Schematic of 250 mL automated suspension culture bioreactor. Dissolved oxygen (DO, 5.25%), pH (7.3 ± 0.2), and temperature (37 °C) are closed‐loop regulated. DO is maintained using a mixture of oxygen, carbon dioxide, and nitrogen injected into the bioreactor headspace. pH is maintained using carbon dioxide and base addition. Mixing rates and hA suspension are controlled by tuning the impeller speed. c) Brightfield images of representative hA aggregates formed under three different impeller speeds: i) *S*_low_, 100–250 RPM ramped impeller speed; ii) *S*_mid_, 200 RPM constant impeller speed; iii) *S*_high_, 200–500 RPM ramped impeller speed. Scale bars, 400 µm. d) Cell density trends for a screen of 15 sets of bioreactor operating parameters, measured over a 5 day culture period. *n* = 1 per screened condition. e) Representative images of i) SCVI‐15 and ii) WTC‐11 hAs on day 3 of culture. Scale bar, 400 µm. Distribution of f) diameters and g) circularity of hAs over three passages of i) SCVI‐15 and ii) WTC‐11 hAs. f) SCVI‐15: P1 = 135.4 (62.2); P2 = 128.1 (54.9); P3 = 131.8 (54.9); WTC‐11: P1 = 173.9 (84.2); P2 = 153.7 (73.2); P3 = 157.4 (69.5) (Median (IQR)). g) SCVI‐15: P1 = 0.82 (0.12); P2 = 0.82 (0.11); P3 = 0.83 (0.10); WTC‐11: P1 = 0.85 (0.07); P2 = 0.84 (0.10); P3 = 0.86 (0.07) (Median (IQR)). f,g) P1, P2, P3 = Passage 1, 2, 3. Median (IQR) shown. SCVI‐15: P1, *n* = 3084 hAs; P2, *n* = 2828 hAs; P3, *n* = 3911 hAs; WTC‐11: P1, *n* = 5674 hAs; P2, *n* = 6333 hAs; P3, *n* = 10488 hAs. h) Flow cytometry analysis of percentage of cell population co‐expressing OCT4, NANOG, SSEA‐4, and TRA‐1‐60. Each point represents the mean measured across 3 technical replicates, *n* = 3 replicates from serial passages. i) Cell density at day 3 of culture. j) Fold expansion of cells calculated from day 0 and day 3 cell densities. k) Viability at day 3 of culture. i–k) *n* = 3 serial passages. Error bars show mean ± s.d. h–k) Key: S: SCVI‐15 (pink), W: WTC‐11 (teal). l) Representative trend lines showing rate of oxygen input.

Here, we develop a scalable bioprinting pipeline, starting with 1) the optimized production and serial passaging of approximately 1 billion hiPSCs in 250 mL automated bioreactors, 2) scaling up production to approximately 4 billion hiPSCs in 1 L suspension cultures, followed by the harvest and compaction of hAs for downstream applications such as 3) rheological characterizations of a wholly‐cellular bioinks composed of compacted hAs or single hiPSCs, and 4) bioprinting and subsequent differentiation of the hAs into different cell types of interest (**Figure** **1**a). By employing iterative wet lab experiments and computational multivariate analyses to refine ideal culture conditions, we characterize the impact of seeding density and initial and final impeller speed on the viable cell density, fold‐expansion, morphology of the hAs at the 250 mL culture scale. In the optimal culture condition, our approach achieved a 23‐fold expansion of cells at a density of 4.89 million cells mL^−1^, while maintaining >94% expression of pluripotency markers. Furthermore, we tuned the impeller speed to obtain hAs with desired diameters ranging from 250 to 300 µm for downstream organoid differentiation or 300–450 µm for serial passaging to facilitate continuous culture. These results were replicated in three serial passages of 3‐day cultures using two hiPSC lines. Subsequently, the culture parameters were scaled to 1 L suspension cultures. Over the 4‐day culture, we achieved 16.6‐ to 20.4‐fold expansion of cells, with densities of 3.61–4.44 million cells mL^−1^, while maintaining >94% expression of pluripotency markers. We showed that hAs from both the 250 mL and 1 L cultures can form organoids originating from the three germ layers via directed differentiation. Finally, we concentrated approximately 4 billion cells from the 1 L bioreactor culture into an hA slurry for rheological characterization, 3D‐bioprinted the hAs into defined shapes, and subsequently differentiated the bioprinted constructs into neuroepithelium or vascular cells. Our work demonstrates a scalable cell manufacturing pipeline for producing hAs, with the potential to scale cell culture to 10 L or larger (billions of cells) for manufacturing wholly cellular bioinks for bioprinting.

## 2 Results

### 2.1 Constant 200 RPM Impeller Speed Yields Highest Proportion of hAs in the Desired Diameter Range

In the first phase to develop a scalable process for producing large quantities of hAs for bioprinting, SCVI‐15 hiPSCs were cultured in the Sartorius Ambr 250 modular bioreactor, a system which enables programmable impeller speeds and real‐time measurements and regulation of temperature, pH, DO, and impeller spin speeds in up to eight individually controllable bioreactors (Figure 1b). hiPSCs were seeded as single cells into the stirred bioreactor and spontaneously aggregated into hAs within one day of seeding. After expansion in the bioreactors using daily batch feeds, hA cultures were harvested for downstream applications.

Automated bioreactors allow for the precise and tunable production of hAs of varying diameters to suit the desired endpoint application. For example, hAs with diameters ranging from 250–350 µm are ideal for cardiomyocyte differentiation,^[^ ^29^, ^30^ ^]^ while hAs with diameters greater than 450 µm must be passaged in order to avoid limitations of oxygen and nutrient diffusion to the core.^[^ ^31^ ^]^ Thus, establishing process parameters to produce consistent hA shapes and diameters and uniform mixing of nutrients and media supplements is critical for an optimized cell manufacturing pipeline. Of the available process parameters, we focused primarily on varying impeller spin speed, as others have found that impeller speed and both impeller and tank geometry can affect mixing rates, hA diameter, and growth.^[^ ^20^, ^32^ ^]^ To systematically identify the optimal impeller spin speeds to produce the largest proportion of hAs with diameters in the 250–450 µm range, we performed a series of Design of Experiment (DoE) runs in the Ambr 250 bioreactor system. In total, 15 sets of impeller spin speed conditions were screened using the SCVI‐15 hiPSC line, with constant or ramped impeller spin speeds (Table S1, Supporting Information). Of the 15 tested conditions, we highlight three different conditions that exemplify the effects of impeller spin speed on the distribution of hA diameters by day 5 of the culture: 1) low initial speed condition, *S*_low_, (100–250 RPM, initial impeller speed at 100 RPM on days 0–1, then linearly ramped up to 250 RPM over days 1–5), 2) constant speed condition, *S*_mid_ (constant at moderate 200 RPM), and 3) high final spin speed conditions, *S*_high_, (200–500 RPM, initial impeller speed at 200 RPM on days 0–1, then linearly ramped up to 500 RPM over days 1–5). For each condition, we sampled hAs daily and measured their size, circularity, and cell density, and on days of harvest we measured pluripotency marker expression. In all cases, hAs increased in size over the culture period (Figure 1d; Figure S1a,b, Supporting Information). Culture time and the initial and final impeller spin speeds strongly affected hA size (Figure 1c; Figures S1c,S2a, Supporting Information). Low spin speeds (as exemplified by the *S*_low_ condition) were associated with excessive aggregation (Figure 1c‐i; Figures S1b‐i,c,S2a, Supporting Information) and lower expression of pluripotency markers (Figure S3, Supporting Information) while high spin speeds, for example, *S*_high_ produced small hAs that were below the size typically recommended for passaging^[^ ^31^ ^]^ (Figure 1c‐iii; Figures S1b‐iii,c, S2a, Supporting Information). In contrast, a moderate and constant spin speed (*S*_mid_) produced hAs with a high level of expression of pluripotency markers (>94% of cells were NANOG+, SSEA‐4+, and TRA‐1‐60+) (Figure S3, Supporting Information), and a median diameter of 245.2 µm and interquartile range of 113.5 µm that is well‐suited for passaging and differentiation (Figure 1c,ii; Figures S1c,S2a, Supporting Information). Pluripotent hAs should have a spherical shape; anisotropy can suggest that the hAs have begun to differentiate,^[^ ^33^ ^]^ are aggregating, or are experiencing high shear conditions. As a proxy for sphericity, we tracked the circularity of brightfield images of hAs, wherein a circularity score of 1 denotes a perfect circle. Overall, the *S*_low_ and *S*_high_ conditions generated hAs with lower circularity due to excessive aggregation and shearing, respectively, while the *S*_mid_ condition resulted in more spherical hAs (Figure S1d, Supporting Information).

Optimizing the yield and pluripotency of hiPSCs are crucial for attaining organ‐scale biofabrication. By day 5, the cell density reached 4–5 million cells mL^−1^ in the *S*_mid_ and *S*_high_ conditions (Figure 1d). The *S*_mid_ condition resulted in a 23‐fold expansion of cells, yielding a total of 4.89 million cells mL^−1^ in 250 mL of culture by day 5 (Figure S1e, Supporting Information). In contrast, cell density was lower (1.02 million cells mL^−1^ by day 5) in the *S*_low_ condition (Figure 1d). The viability is greater than 95% on days 2–5 for all three impeller speed conditions, with the exception of *S*_low_ where excessive aggregation by day 5 results in a lower viability (Figure S1f, Supporting Information). Of note, the cell viability is low on day 1 in all conditions likely because of cell death associated with single cell hiPSC passaging.^[^ ^34^ ^]^ Media changes from day 1 onward remove single dead cells from the media, resulting in a higher measured cell viability for days 2–5 (Figure S1f, Supporting Information).

The bioreactor can track several operational parameters in real‐time, such as the volume of oxygen pumped into the system to fuel metabolism, or the volume of base added to the system to offset acidic metabolic byproducts. These real‐time measurements can be used as surrogate measures for monitoring cell proliferation. For example, the rate of oxygen input required to maintain the DO setpoint in the *S*_mid_ condition increased the most markedly over time, consistent with this condition producing the highest cell yield by day 5 (Figure 1d; Figure S1g, Supporting Information). Despite having similar cell density measurements at each timepoint, the rate of oxygen input in the *S*_high_ condition was lower than the *S*_mid_ condition during days 3–5. This is likely due to the higher impeller speed in the *S*_high_ condition increasing the diffusion rate of oxygen from the headspace into the media. The rate of oxygen input for the *S*_low_ condition had irregular spikes between days 0 and 1, likely due to large aggregates occluding the light‐based DO sensor. These spikes were mitigated by the start of day 1 when the impeller speed ramped up (Figure S1g, Supporting Information). In addition to this, the rate of base (1.0 m sodium bicarbonate) addition can also be used as a surrogate measure of cell density and metabolism (Figure S1h, Supporting Information). As cells respire and release carbon dioxide and other acidic metabolites into culture media, the pH of the media decreases. In our closed‐loop control system, base is added to maintain pH at the ideal setpoint for the media. In the *S*_mid_ and *S*_high_ conditions, base addition began on day 3 to maintain the desired pH range of 7.3 ± 0.2. The rate of base addition ceases momentarily after every media refresh on day 3, 4, and 5, and its input is triggered only after carbon dioxide and other acidic metabolites reach a certain threshold concentration in the media again (Figure S1h, Supporting Information, *S*_mid_: black line, *S*_high_: teal line). In the *S*_low_ conditions, the cell density is insufficiently high throughout 5 days of culture, and no base addition was required throughout the culture (Figure S1h, pink line, Supporting Information).

To identify the optimal impeller speed parameters, we conducted a post hoc multivariate data analysis (MVDA) on all 15 conditions tested in the DoE. The algorithm returned the range of impeller speeds predicted to produce hAs with the desired characteristics, including a cell density >1 million cells mL^−1^, the largest proportion of hAs with diameters of 251–450 µm, >90% expression of pluripotency markers NANOG, SSEA‐4, and TRA‐1‐60, and >0.80 circularity (Table S2, Supporting Information). The analysis returned the range of impeller speeds predicted to yield the largest percentage of hAs with diameters in the 251–450 µm range (Table S3 and Figure S4, Supporting Information), while also maintaining the aforementioned desired characteristics. The *S*_mid_ condition (200 RPM constant speed) was within this optimal range. We had hypothesized that ramping up the impeller speed would be necessary to keep the hAs suspended as they increased in diameter. However, the MVDA results indicated that moderately increasing impeller speeds over time had negligible effects on hA diameter distributions and excessively high final impeller speeds, up to 500 RPM, led to smaller hA diameters, likely due to excessive shear stress. Furthermore, given that *S*_mid_ not only fulfilled our MVDA criteria, but also resulted in the highest cell density (Figure 1d) among all experimental runs, we selected the *S*_mid_ condition for subsequent experiments.

### 2.2 hiPSC Aggregates Maintain Growth Rates and High Pluripotency When Cultured for Multiple Passages in Bioreactors

A robust and continuous pipeline of hA culture is required to ensure an adequate supply of starting material for bioprinting and differentiation applications, as well as sufficiently pluripotent hAs for ongoing passaging and expansion. Thus, to demonstrate the viability of a continuous hA production pipeline, we conducted a run of three serial passages wherein new cultures were inoculated using hAs grown from the previous culture. For each passage, a portion of the hA culture was harvested for bioprinting and/or differentiation into desired cell types, and the remaining hAs were dissociated into single cells and reseeded into another bioreactor. To demonstrate that the *S*_mid_ parameters could be applied to different hiPSC lines, we performed our tests using WTC‐11 and SCVI‐15 lines. Both lines were karyotypically normal when grown as adherent cultures (Figure S5a,b, Supporting Information). We harvested hAs on day 3 to maximize the percentage of hAs with diameters ≤ 300 µm, which has been reported to be more favorable for efficient differentiation into multiple cell types.^[^ ^29^, ^30^, ^31^ ^]^ 76.0 ± 4.2% (mean ± SD) of SCVI‐15 and 93.7 ± 3.4% WTC‐11 hAs had diameters ≤ 300 µm (Figure 1e,f; Figure S2b, Supporting Information). At least 75% of hAs in both cell lines had a circularity of 0.73 or higher (Figure 1g). Within the three serial passages of each cell line, the distribution of hA diameters and circularity was consistent (Figure 1f,g). On day 3, 95.4 ± 2.0% (mean ± SD) of SCVI‐15 cells and 90.0 ± 5.5% of WTC‐11 cells expressed OCT4, NANOG, SSEA‐4, and TRA‐1‐60 (Figure 1h). Furthermore, the cell density of SCVI‐15 cells was 1.81 ± 0.49 million cells mL^−1^ representing a 10.3 ± 4.6‐fold expansion and the cell density of WTC‐11 cells was 2.29 ± 0.81 million cells mL^−1^, representing a 11.3 ± 4.2‐fold expansion (Figure 1i,j). SCVI‐15 cells were 89.7 ± 5.0% viable and WTC‐11 cells were 91.8 ± 3.1 viable (Figure 1k). The rate of oxygen input also increased as cell density increased (Figure 1l). Immunofluorescence images also showed that OCT4, NANOG, and TRA‐1‐60 were expressed uniformly throughout the hAs (Figure S6, Supporting Information). After serial passaging, WTC‐11 cells were karyotypically normal (Figure S5c, Supporting Information). In contrast, a proportion of the SCVI‐15 cells tested (5 out of 20 cells) contained an unbalanced rearrangement of chromosome 1 in which portions of the long (q) arm of chromosome 1 were duplicated (Figure S5d, Supporting Information). Given that 1q duplications are known to be recurrent in hiPSC cultures and are associated with a proliferative advantage,^[^ ^35^ ^]^ these cells may have increased in proportion over multiple passages. Taken together, our results show that hAs can be robustly and serially passaged in bioreactors, producing hAs with consistent growth rates and morphologies while maintaining high rates of pluripotency.

### 2.3 hiPSC Ag Culture can be Scaled to 1 L

**Figure 2:**
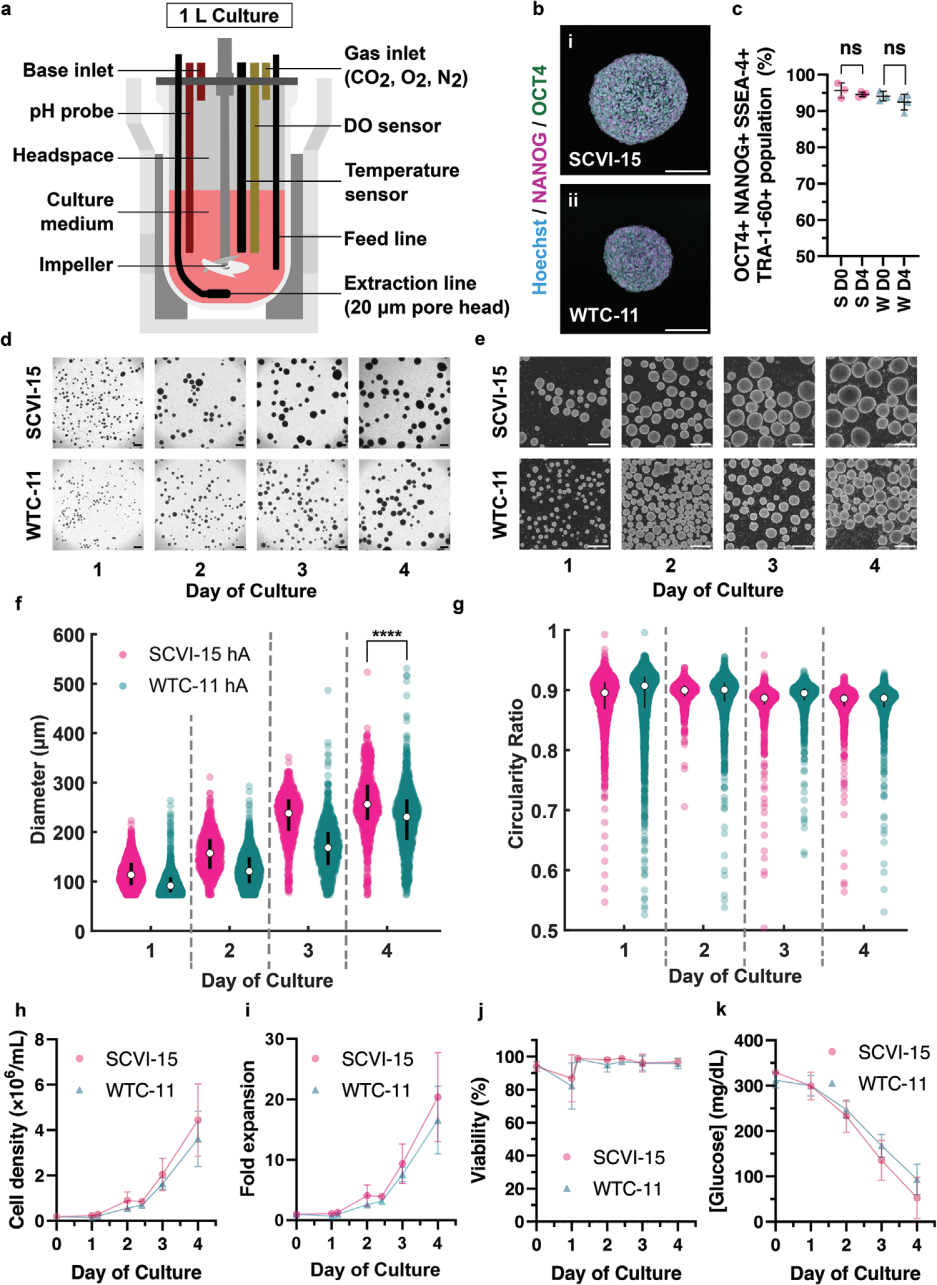
Scaling‐up to 1L bioreactors to produce large quantities of hiPSC aggregates. a) Schematic of the BioStat B‐DCU and UniVessel 2 L automated bioreactor. Dissolved oxygen (DO, 5.25%), pH (7.3 ± 0.2), and temperature (37 °C) are closed‐loop regulated. The impeller speed is set at 113 RPM for the duration of the culture. Medium is exchanged at 1 vessel volume per day (VVD) under a weight control loop, where old medium is extracted through a 20 µm pore frit, and fresh medium is pumped in via a feed line. b) Immunofluorescence staining of day 4 i) SCVI‐15 and ii) WTC‐11 hAs for NANOG (magenta), OCT4 (green), and Hoechst (cyan). Scale bars, 100 µm. c) Flow cytometry analysis showing the percent of cells co‐expressing OCT4, NANOG, SSEA‐4, and TRA‐1‐60 pluripotency markers. Key: S: SCVI‐15 (pink), W: WTC‐11 (teal), D0: Day 0, D4: Day 4. Each point represents the mean pluripotency marker percent expression across 3 technical replicates, *n* = 3–4 biological replicates per condition. ns: not significant, unpaired two‐tailed Student's *t*‐test. Error bars show mean ± s.d. d) 4× and e) 10× brightfield images of hAs sampled from the bioreactor on days 1–4. Scale bars, 400 µm. f) Distribution of diameters of SCVI‐15 (pink) and WTC‐11 (teal) hAs. Error bars show median, IQR. *n* = 660 – 4164 hAs per condition, aggregated from *n* = 4 replicates for SCVI‐15, and *n* = 3 replicates for WTC‐11. ****p <0.0001, Mann–Whitney U‐test. g) Circularity scores of hAs. Bars show median, IQR. *n* = 660–4164 hAs per condition aggregated from *n* = 3 replicates per cell line. h) Cell density, i) fold expansion, j) viability, and k) glucose concentration during the course of the hA culture. h–k) Error bars show mean ± s.d., *n* = 3 biological replicates per condition.

In an hA bioink production pipeline, billions of cells are needed to conduct organ‐scale prints. We achieved a total yield of approximately 1 billion cells per 250 mL of culture media in the Ambr 250 modular system. As a next step, to increase the number of cells generated and to demonstrate the scalability of this process, we next conducted 1 L cultures of SCVI‐15 and WTC‐11 hAs. To carry out the 1 L cultures over a 4‐day period, we utilized the Sartorius BioStat B‐DCU automated bioreactor system. Similar to the Ambr 250 modular system, the BioStat enables programmable impeller speeds and real‐time measurements and regulation of temperature, pH, and DO in up to 2 bioreactors (**Figure** **2**a). We scaled the optimal impeller spin speed identified in the Ambr 250 modular system in Figure 1 by calculating an equivalent tip speed in the 2 L BioStat Univessel (operating at 1 L culture volume). Furthermore, we also implemented continuous, perfused feeding of fresh medium into the bioreactor starting at day 1 of the experiment, as opposed to the daily batch feeding used previously in the Ambr 250 modular system, whereby nearly an entire reactor volume is exchanged at once daily. Notably, others have found perfusion feeding increases yield by avoiding nutrient depletion through continuous media exchange.^[^ ^20^ ^]^ Moreover, perfusion feeding also lends to automating the culture process, as no manual intervention is needed to maintain the culture past day 1, after the perfusion system is set up. All other conditions, including seeding density, pH setpoint, DO setpoint, and temperature setpoint, were kept constant. Similar to the hAs in the Ambr 250 modular system, both SCVI‐15 and WTC‐11 hAs sampled at the end of the culture period expressed pluripotency markers NANOG and OCT4 throughout the hA (Figure 2b). Flow cytometry analysis showed that 95.7 ± 2.1% of SCVI‐15 and 94.1 ± 1.3% of WTC‐11 cells inoculated (day 0) co‐expressed pluripotency markers OCT4, NANOG, SSEA‐4, and TRA‐1‐60 and maintained high levels of pluripotency expression after 4 days in culture in the bioreactor (SCVI‐15 94.6 ± 0.6%, WTC‐11 92.5 ± 2.2%, Figure 2c, *n* = 3 replicates per cell line, mean ± s.d.). Similar to the Ambr 250 modular system, hAs sampled from the 1 L bioreactor increased in diameter over time (Figure 2d,e). Median diameters at day 4 were 256.2 (65.9) µm for SCVI‐15 hAs and 230.6 (76.8) µm for WTC‐11 cells (Figure 2f). Additionally, at least 98% of hAs had circularity scores higher than 0.73, indicating low hA‐hA aggregation and shearing (Figure 2g). On day 4, the cell density of SCVI‐15 cells was 4.44 ± 1.60 million cells mL^−1^ (20.4 ± 7.3‐fold expansion) and WTC‐11 cells was 3.61 ± 1.22 cells mL^−1^ (16.6 ± 5.6‐fold expansion) (Figure 2h,i). Across the 4 day culture period, SCVI‐15 cells and WTC‐11 cells were 95.0 ± 6.6% and 93.8 ± 7.3% viable (mean ± s.d. across all days), respectively (Figure 2j). Glucose concentration in the media can be a readout of the relative amount of cell growth, with lower concentrations indicating high glucose consumption from glycolysis. Thus, we measured glucose concentrations in the media daily, and found that with increasing cell density, the glucose concentrations decreased with the duration of culture (Figure 2k). After 4 days of culture, WTC‐11 cells had a normal karyotype (Figure S5e, Supporting Information). In contrast, a proportion of the SCVI‐15 cells tested (8 out of 20) had portions of the long (q) arm of chromosome 1 that were duplicated (Figure S5f, Supporting Information). Given that a proportion of the SCVI‐15 hiPSCs cultured in the Ambr 250 modular and BioStat B‐DCU bioreactors are biologically independent cultures which originated from the same stocks, and yet contain identical 1q duplications, it is possible that the karyotypic abnormalities found in the SCVI‐15 hiPSCs may be a result of a preferential expansion of a pre‐existing subpopulation of cells bearing the 1q duplication in the stocks. Taken together, our results show that hA culture can be robustly scaled up to 1 L with consistent growth rates and morphologies while maintaining high rates of pluripotency.

### 2.4 hiPSC Aggregates Differentiate into Organoids of Ectodermal, Mesodermal, or Endodermal Origins

**Figure 3:**
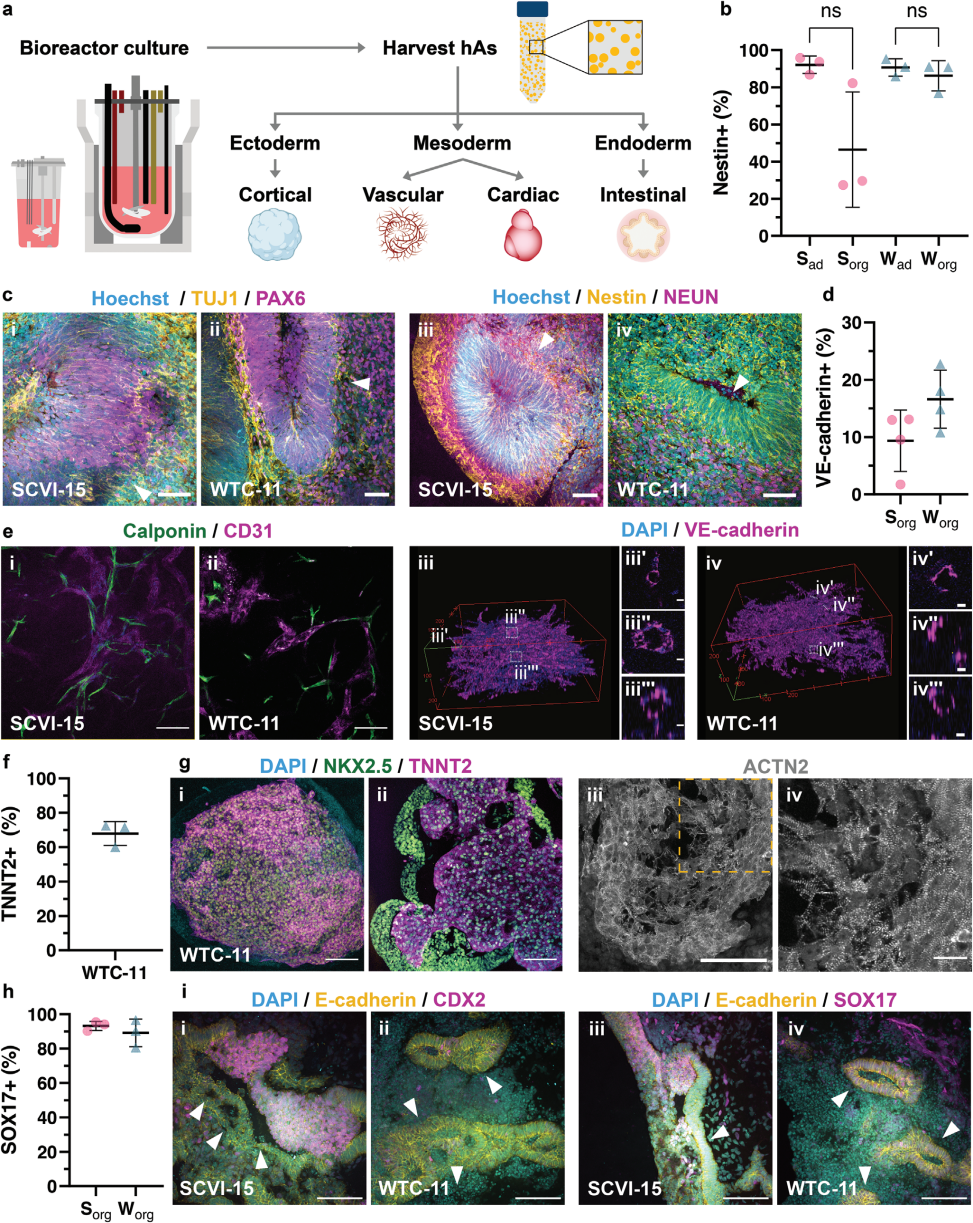
Characterizations of differentiated organoids derived from SCVI‐15 and WTC‐11 hAs. a) Schematic of workflow for generating hAs in bioreactors and subsequently differentiating them into aggregates or organoids of each germ layer. b) Flow cytometry analysis of the percentage of Nestin+ cells in day 10 neuroepithelium suspension cultures (“org”) and day 9 adherent (“ad”) neuroepithelium cultures for SCVI‐15 (“s”) and WTC‐11 (“w”) hAs. ns: not significant, unpaired two‐tailed *t*‐test. *n* = 3 biological replicates per condition. Error bars show mean ± s.d. c) Cryosection of day 45 cortical organoids. i,ii) Organoids stained for TUJ1 (yellow), PAX6 (magenta), and Hoechst (cyan). iii,iv) Organoids stained for Nestin (yellow), NEUN (magenta), and Hoechst (cyan). White arrows indicate ventricle‐like structures. Scale bars, 50 µm. d) Flow cytometry analysis of the percentage of VE‐cadherin+ cells in day 10 vascular organoids. *n* = 4 replicates. Bars show mean ± SD. e) Wholemount IF of day 10 i) SCVI‐15 and ii) WTC‐11 derived vascular organoids stained for Calponin 1 (CNN1) (green) and CD31 (magenta). Scale bar, 100 µm. 3D render of wholemount IF, day 10 iii) SCVI‐15 and iv) WTC‐11 vascular organoids stained for VE‐cadherin (magenta) and DAPI (blue). Insets (iii′–iii''′, iv'‐iv''') show lumen‐like structures within the vascular organoid network. Red boxes (iii, iv) 500 µm across; inset scale bars (iii′–iii″′, iv′–iv″′) 10 µm. f) Flow cytometry analysis of percentage of cardiac Troponin T (TNNT2)+ cells in day 9 hA‐derived cardiac aggregates. Bars show mean ± s.d., *n* = 3 replicates. g) Day 10 i) wholemount or ii) cryosectioned hA‐derived cardiac aggregate stained for NKX2.5 (green), TNNT2 (magenta), and DAPI (blue). iii) Cryosectioned organoid stained for *α*‐actinin (ACTN2). iv) Dashed box shows the region of interest. Scale bars, i–iii) 100 µm, iv) 25 µm. h) Flow cytometry analysis of the percentage of SOX17+ cells in day 3 definitive endoderm cells. *n* = 3 replicates. Bars show mean ± s.d. i) Cryosection of day 15 intestinal organoids stained for i,ii) E‐cadherin (yellow), CDX2 (magenta), and DAPI (cyan), and iii,iv) E‐cadherin (yellow), SOX17 (magenta), and DAPI (cyan). White arrows indicate lumen‐like structures. Scale bar, 100 µm. b,d,f,h) pink: SCVI‐15, teal: WTC‐11. Differentiated organoids used in the flow cytometry analysis shown in (b,d, f, h) were derived from hAs cultured in the 1 L BioStat bioreactor. Immunofluorescence images are of organoids derived from hAs cultured in the 250 mL Ambr bioreactor. Identical differentiation protocols were used in all differentiations.

Having established that hAs can be maintained in suspension culture over multiple passages and that process parameters can be scaled up to produce a larger number of hAs, we next assessed their potential to differentiate into organoids at each passage (**Figure** **3**). Specifically, we directed the differentiation of hAs to produce organoids or differentiated cell aggregates of ectodermal, mesodermal and endodermal origins, using hAs derived from SCVI‐15 and WTC‐11 cells cultured in the Ambr 250 and 1 L BioStat (Figure 3a). We harvested and passaged hAs on day 3 (Ambr 250) or day 4 (BioStat) to maximize the number of cells in hAs which are less than 300 µm in diameter, because hAs within the 250–300 µm range have been shown to have the highest efficiency for downstream differentiation.^[^ ^29^, ^30^, ^31^ ^]^ These SCVI‐15 and WTC‐11 hAs were differentiated into cortical organoids using previously described protocols.^[^ ^36^, ^37^, ^38^, ^39^ ^]^ On day 10 of the differentiation into neuroepithelium, 46.4 ± 31% (mean ± SD) of the SCVI‐15 and 86.2 ± 8.1% (mean ± SD) of the WTC‐11 cells expressed early neuronal marker Nestin (Figure 3b). For each cell line, the Nestin expression in the aggregates were not significantly different when compared to differentiations on 2D cells (SCVI‐15 2D: 92.1 ± 4.5%, mean ± SD, WTC‐11 2D 90.7 ± 4.7%, mean ± s.d., not significant, unpaired *t*‐test). By day 45 of the differentiation, the organoids expressed neuroepithelial markers PAX6 and Nestin, and neuronal markers, TUJ1 and NEUN (Figure 3c). Specifically, TUJ1 and Nestin expression patterns reveal characteristic morphologies present in cortical organoids (Figure 3c). Furthermore, regions of the cortical organoids self‐assembled into ventricle‐like structures (Figure 3c, white arrowheads).^[^ ^38^ ^]^

To validate that SCVI‐15 and WTC‐11 hAs could differentiate into mesodermal tissues, we differentiated a sample of hAs into vascular organoids using a previously established method.^[^ ^40^ ^]^ At day 10 of differentiation, 9.4 ± 5.3% (mean ± SD) of SCVI‐15 and 16.6 ± 5.1% (mean ± SD) of WTC‐11 cells expressed vascular endothelial cell marker VE‐cadherin (Figure 3d). Furthermore, SCVI‐15 and WTC‐11 hAs formed vascular networks containing Calponin+ mesenchymal progenitor cells and CD31+ vascular endothelial cells (Figure 3e‐i,ii). Additionally, the differentiated hAs also formed microvascular networks, with lumen‐like structures (Figure 3e‐iii,iv; Movie S1, Supporting Information). In addition to the vascular differentiations, we also differentiated WTC‐11 hAs into cardiac aggregates, which showed spontaneous contraction by day 7 (Movie S2, Supporting Information). On day 9, 68.0 ± 7.0% (mean ± SD) of WTC‐11 cells expressed cardiomyocyte marker cardiac Troponin T (TNNT2) (Figure 3f). By day 10, these cardiac aggregates expressed the cardiomyocyte markers TNNT2 and NKX2.5 (Figure 3g,i) and a cross‐section revealed chamber‐like features (Figure 3g‐ii) and *α*‐actinin+ (ACTN2) sarcomeric structures (Figure 3g‐iii,iv), all of which are key cardiomyocyte morphological features.^[^ ^41^ ^]^

We also differentiated SCVI‐15 and WTC‐11 hAs into endodermal‐origin intestinal organoids using an adapted version of a previously described protocol.^[^ ^42^ ^]^ At day 3 of the differentiation, 93.2 ± 2.7% of SCVI‐15 and 89.1± 8% of WTC‐11 cells expressed definitive endoderm marker SOX17 (Figure 3h). By day 15 of the differentiation, E‐cadherin expression revealed lumen‐like architectures bound by polarized epithelium (Figure 3i, white arrows). Furthermore, these organoids contained regions of CDX2+ intestinal progenitors (Figure 3i‐i,ii) and SOX17+ definitive endodermal tissues (Figure 3i‐iii,iv). Taken together, these results show that hAs cultured in the bioreactors over several passages retained the potential to form organoids representing derivatives of ectodermal, mesodermal, or endodermal lineages.

### 2.5 hiPSC Aggregate Bioinks Exhibit A Viscoelastic, Thixotropic, Yield‐Stress, and Self‐Healing Rheology

**Figure 4:**
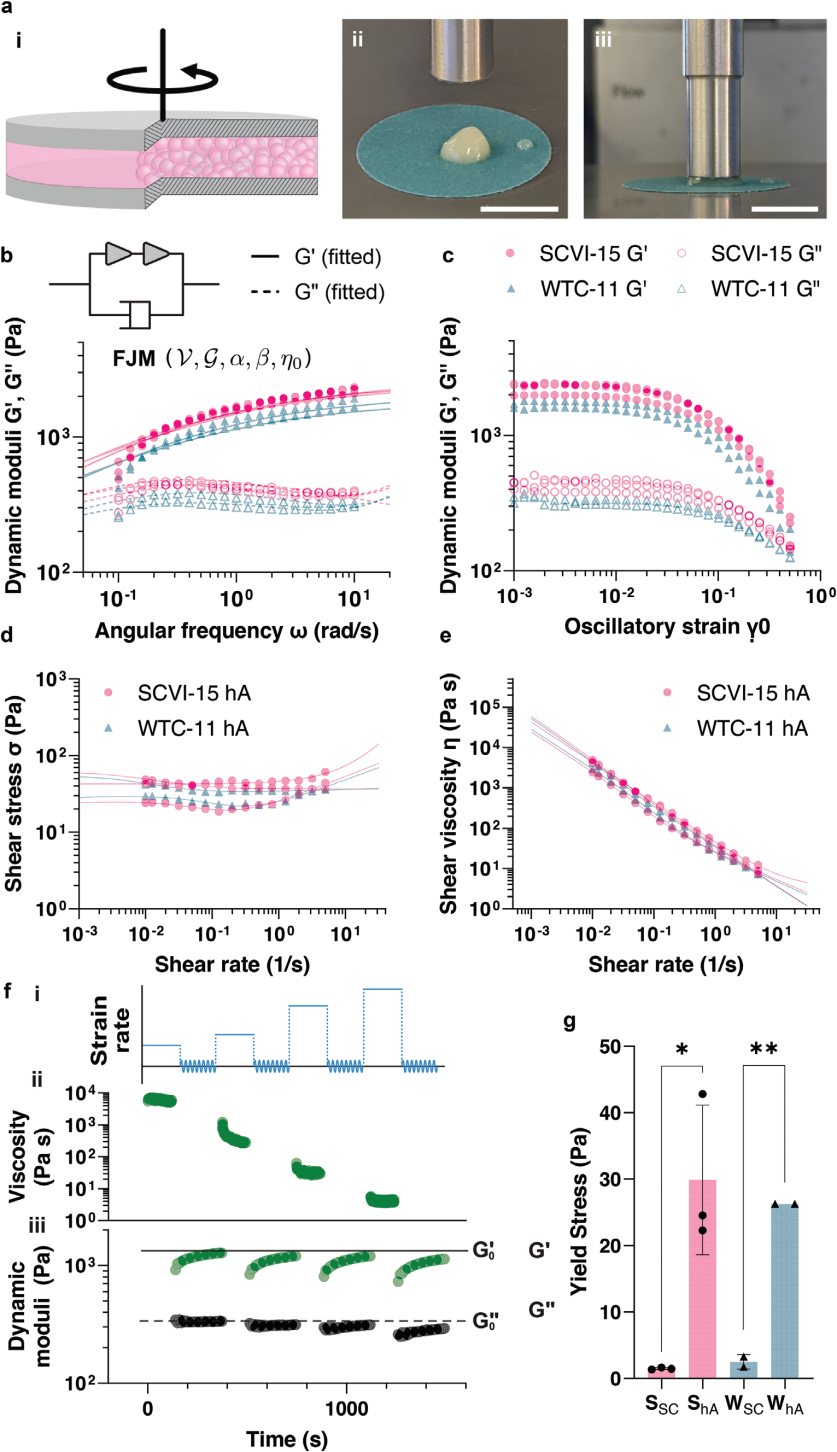
Rheological characterization of hA bioinks. a) Rheological measurement setup. i) Schematic of the applied plate‐plate geometry loaded with a sectional view showing dense slurries of hAs (pink). ii,iii) hA bioinks loaded onto a sandpaper‐coated substrate (grit number 320), ii) before and iii) after an 8 mm plate is brought to the specified experimental gap. Scale bar, 10 mm. b) Frequency response of SCVI‐15 (pink) and WTC‐11 (teal) hA bioinks measured at 0.1–10 rad s^−1^ at a fixed amplitude of 1%. Filled circles and triangles are storage moduli values and empty circles and triangles are loss moduli values. Solid (storage moduli) and dashed (loss moduli) lines are fit using a fractional Jeffrey model (FJM). Inset: Component diagram of FJM used to fit the linear viscoelastic responses of SCVI‐15 and WTC hA bioinks (see Section 4). c) Amplitude response for SCVI‐15 and WTC‐11 hA bioinks measured at 10^−4^ to 0.5 at a fixed frequency of 0.5 Hz. Filled circles and triangles are storage moduli values and empty circles and triangles are loss moduli values. d) Steady shear stress and e) viscosity plotted against shear rates (0.01–5 s^−1^) for SCVI‐15 (pink circles) and WTC‐11 (teal triangles) hAs. Solid lines are fitting lines from the modified Herschel–Bulkley model with thixotropic contributions (Equation (1)). f) Flow‐recovery test for SCVI‐15 hAs at varying strain rates. i) Schematic of input strain rate with alternating steady shear motion and small‐amplitude oscillation. ii) Transient viscosity during the peak‐hold tests at strain rates of 0.01 s^−1^, 0.1 s^−1^, 1 s^−1^, and 10 s^−1^, respectively. iii) Transient dynamic moduli upon the flow cessation of the aforementioned peak‐hold tests performed at a frequency of 1 rad/s and amplitude of 1%. g) Yield stresses of SCVI‐15 and WTC‐11 hAs and single cells (SCs), extracted from fitting the modified Herschel–Bulkley model to the corresponding flow curves. SCVI‐15 hAs and SCs, *n* = 3; WTC‐11 hAs and SCs, *n* = 2, mean ± s.d. *p < 0.05, **p < 0.01, unpaired two‐tailed Student's *t*‐test. All measurements are performed at 20 °C.

To ensure a high degree of printability, our wholly cellular bioinks should exhibit a shear thinning and yield stress rheology with fast healing dynamics after printing.^[^ ^43^ ^]^ Notably, shear stresses should be minimized during bioprinting to ensure cell viability.^[^ ^44^ ^]^ Culturing hAs at the 1 L‐scale permits the generation of billions of cells to render large quantities of wholly cellular bioink for detailed rheological analysis. To this end, we compacted SCVI‐15 and WTC‐11 hAs into a jammed and granular bioink and measured their rheological properties (**Figure** **4**a). The SCVI‐15 and WTC‐11 hA bioinks showed similar viscoelastic frequency and amplitude responses (Figure 4b,c). The storage modulus, *G*′ (filled markers), for both samples increased with frequency, while the loss modulus, *G*″ (hollow markers), remained broadly constant, with *G*′ >> *G*″ across measured frequencies (i.e., tan *δ* = *G*″/*G*′ << 1), indicating dominant elastic behavior for both hA bioinks (Figure 4b). The linear viscoelastic behavior for both slurries notably deviated from Maxwellian dynamics, and can be well‐fit by a fractional Jeffrey model (FJM), which exhibits more complex fractional‐power‐law dynamics^[^ ^45^ ^]^ (see Section 4). Such a power‐law relation suggests that contributions to the rheology arise broadly from multiscale interactions,^[^ ^46^, ^47^ ^]^ that is, at the sub‐cellular, cellular, and hA scales. In the FJM, multiscale contributions to the complex viscoelastic responses can be readily described by a compact constitutive relation using fractional calculus, visualized as two spring‐pots (combination of spring and dashpot; see inset of Figure 4b) connected in series.^[^ ^45^ ^]^ Another dashpot connected in parallel with the two spring‐pots is added to describe the contributions by non‐cellular, residual media present in the bioink, which may only become prominent at high oscillating frequencies. The amplitude response probed at a fixed frequency of 0.5 Hz showed that all hA bioinks yielded at a critical strain of approximately 3% (Figure 4c). The hA slurries inspected under steady‐shear conditions at varying strain rates and corrected for parallel‐plate geometry^[^ ^38^ ^]^ showed non‐trivial yield stresses (Figure 4d) and strong shear‐thinning behavior (Figure 4e). Particularly, we noticed a non‐monotonic trend in the steady‐state stress curves with varying strain rates. To better understand the origin of this complex flow behavior, we performed flow‐recovery cycles at varying shear rates on SCVI‐15 hAs (Figure 4f). A decreasing transient viscosity (Figure 4f,ii) is captured in each peak‐hold cycle and the viscoelasticity upon flow cessation recovers consistently to the original state in an exponentially‐decaying dynamic (Figure S7, Supporting Information), both of which are clear evidence of thixotropic behavior. Thus, to extract accurate constitutive parameters from the shear‐dependent material responses, we modified the Herschel–Bulkley model^[^ ^48^ ^]^ with the addition of thixotropic contributions as:

(1) $$\sigma =\frac{\sigma_{0}}{1+\lambda \dot{\gamma }}+K{\dot{\gamma }}^{n}$$

where *σ*_0_ is the shear‐independent intrinsic yield stress at intact state, and *K* and *n* are empirical parameters to describe the power‐law behavior at high shear rates. A new timescale *λ* is introduced to describe the rate of structural reconstruction due to spontaneous and collective microscopic motion, which counteracts the shear‐induced destruction. When *λ* is positive, the steady stress will exhibit a non‐monotonic trend as the strain rate varies (see Supporting Information for the derivation of Equation (1)).

By fitting Equation (1) into the experimental data (solid lines in Figure 4d,e, where the viscosity $\eta =\sigma /\dot{\gamma }$), we extracted the intrinsic yield stresses for SCVI‐15 and WTC‐11 hAs and single cells (Figure S8, Supporting Information). We found that the yield stresses of SCVI‐15 and WTC‐11 hAs (29.9 Pa and 26.3 Pa) are significantly higher than those of single hiPSCs (1.5 Pa and 2.5 Pa) (Figure 4g, *p* < 0.05). As the yield stress of a bioink increases, the size limit and shape fidelity of a biofabricated construct are improved due to enhanced self‐supporting capabilities. Therefore, the hA bioinks hold promise for fabricating larger and more complex tissue constructs.

### 2.6 Compacted hA Bioinks Can Be Bioprinted and Differentiated into Vascular and Neuroepithelial Tissues

**Figure 5:**
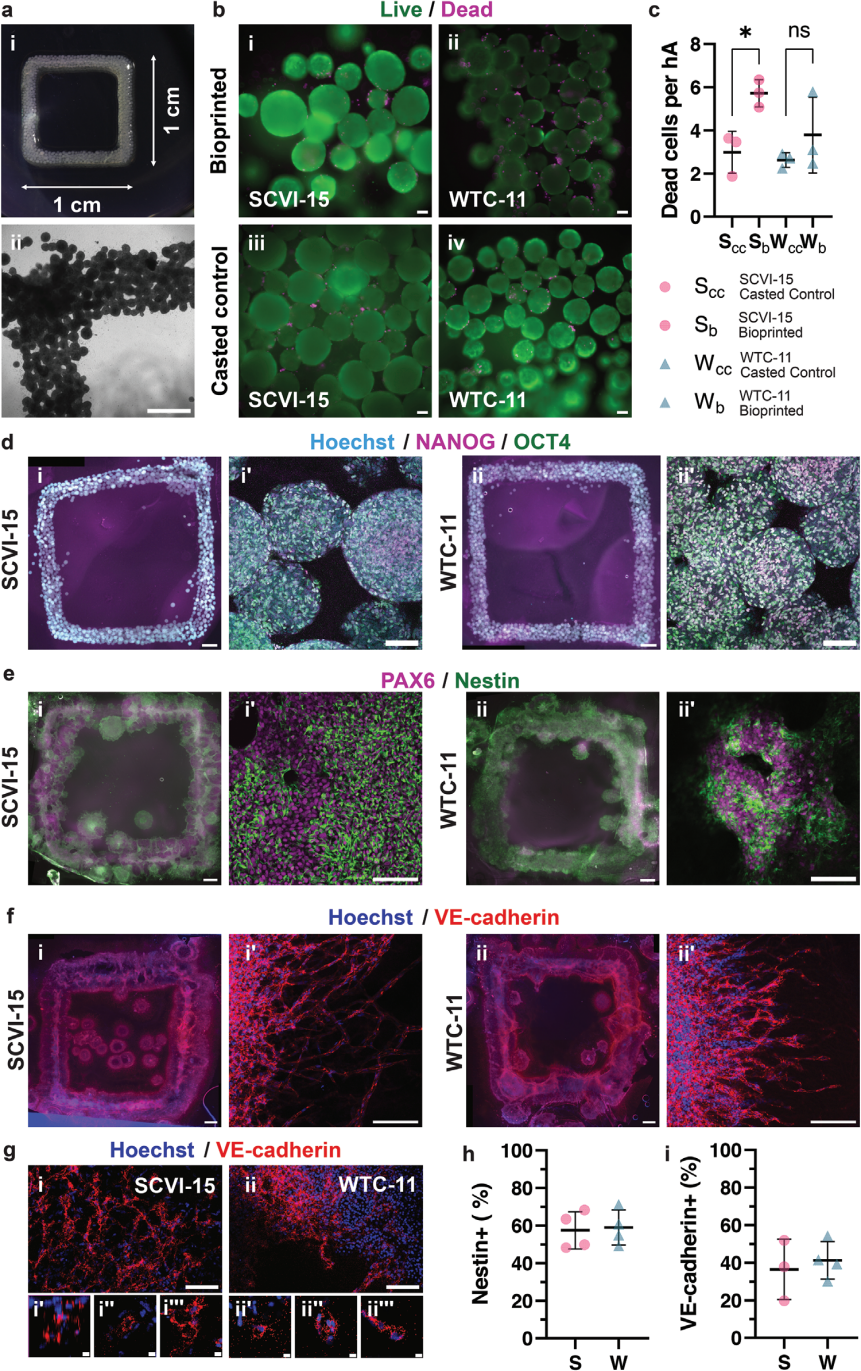
Bioprinting and subsequent differentiation of hA‐based wholly cellular bioinks. a‐i) Top view of day 0 bioprinted hA‐bioink embedded in a collagen‐Matrigel matrix. Square dimensions are approximately 1 cm × 1 cm × 0.1 cm; each arm is 1 mm in width. ii) Brightfield image of a corner of a bioprinted square at day 0. Scale bar, 1 mm. b) Viability of hAs in day 0 bioprinted squares was analyzed using a live/dead stain (green, live cells; magenta, dead cells). Bioprinted i) SCVI‐15 or ii) WTC‐11 hA bioinks; casted control iii) SCVI‐15 and iv) WTC‐11 hA‐bioinks. Scale bars, 100 µm. c) Dead cells per hA after bioprinting. *S*_cc_ = SCVI‐15 casted control, *S*_b_ = SCVI‐15 bioprinted, *W*_cc_ = WTC‐11 casted control, *W*_b_ = WTC‐11 bioprinted. Each point represents an average number of dead cells divided by the number of hAs counted across 4–5 fields of view (>100 hAs per data point). *n* = 3 biological replicates per condition. Bars show mean ± s.d. *p < 0.05, ns = not significant, unpaired two‐tailed Student's *t*‐test with Welch's correction. d) Day 0 i) SCVI‐15 and ii) WTC‐11 bioprinted squares immunostained for pluripotency markers NANOG (magenta), OCT4 (green), and Hoechst (cyan). Scale bars, 1000 µm. Maximum intensity Z‐projections showing detailed view of bioprinted i′) SCVI‐15 and ii′) WTC‐11 hAs. Scale bars, 100 µm. e) Day 10 neuroepithelium bioprinted i) SCVI‐15 and ii) WTC‐11 squares immunostained for Nestin (green) and PAX6 (magenta). Scale bars, 1000 µm. Detailed views of day 10 neuroepithelium bioprinted i′) SCVI‐15 and ii″) WTC‐11 hAs. Scale bars, 100 µm. f) Day 10 vascular bioprinted i) SCVI‐15 and ii) WTC‐11 squares immunostained for VE‐cadherin (red) and Hoechst (blue). Scale bars, 1000 µm. Detailed views of day 10 vascular bioprinted i′) SCVI‐15 and ii″) WTC‐11 cells with VE‐cadherin+ angiogenic sprouts. Scale bars, 100 µm. g) Networks of VE‐cadherin+ cells present in day 10 vascular bioprinted squares (red, VE‐caherin; blue, Hoechst). i) SCVI‐15, ii) WTC‐11. Scale bar, 100 µm. i′–i″′,ii′–ii″′) Higher magnification of VE‐cadherin+ cellular networks. Scale bars 10 µm. h) Flow cytometry analysis of Nestin+ cells in day 10 neuroepithelium squares (pink, SCVI‐15; teal, WTC‐11), *n* = 4 biological replicates per condition, bars show mean ± s.d. i) Flow cytometry analysis of VE‐cadherin+ cells in day 10 vascular squares (pink, SCVI‐15; teal, WTC‐11). SCVI‐15 *n* = 3; WTC‐11 *n* = 4 biological replicates per condition, bars show mean ± s.d.

To demonstrate the printability of bioreactor‐derived hAs, the hA bioink was 3D printed in an arbitrary shape into a partially gelled collagen‐Matrigel mixture, which serves as a support bath^[^ ^49^ ^]^ (Movie S3, Supporting Information). The shape of the printed construct was preserved while the tissue and matrix were transferred to 37 °C to drive further gelation of the matrix (**Figure** **5**a). The hAs were held in a densely packed manner post‐printing (Figure 5a‐ii). Compared with previous studies that use wholly‐cellular bioinks to fabricate complex geometries,^[^ ^50^, ^51^ ^]^ the shape fidelity of the tissue construct made from our compacted hA bioinks is comparable. However, the shape fidelity of tissue constructs fabricated from wholly‐cellular bioinks, which have a much higher cell density, larger aggregate size, and lower yield stress, is lower when compared with those from scaffold bioinks. Nevertheless, the high cellular density in wholly‐cellular bioinks offers great promise for manufacturing densely cellular tissues.^[^ ^52^ ^]^ From the rheology data captured (Section 2.5), we predict that the hAs were subject to shear stresses below 100 Pa as they flowed through the tapered nozzle for a time period of <10 s. This low shear stress resulted in a high post‐printing viability, with 5.7 ± 1.7 (mean ± s.d.) dead cells/hA for the SCVI‐15 hA bioink, and 3.8 ± 1.9 (mean ± s.d.) dead cells/hA for the WTC‐11 hA bioink, representing 1% or fewer of the number of cells in each hA (Figure 5b,c), which is comparable to other studies using scaffold and scaffold‐free bioinks.^[^ ^50^, ^51^ ^]^ The viability of the hAs extruded through a narrow nozzle, as measured using a commercially available live/dead stain immediately after printing, was comparable to or moderately less than that of hAs extruded through a syringe with no nozzle attached (casted control: 2.9 ± 1.5 dead cells/hA for SCVI‐15 hAs and 2.6 ± 0.8 dead cells/hA for WTC‐11 hAs, mean ± s.d., *p* < 0.05 for SCVI‐15 hAs and not significant for WTC‐11 hAs). This suggests that the shear stresses imparted to the hAs during extrusion through the nozzle have minimal effects on cell viability and that printing parameters may be tailored for each cell line and/or according to median hA size. Specifically, on day 4 of culture, the diameters of SCVI‐15 hAs were 1 256.2 (65.9) µm (median, [IQR]), which was significantly larger than that of WTC‐11 hAs, which were 230.6 (76.8)  µm (median, [IQR]) (Mann–Whitney U‐test, *p* < 0.0001) (Figure 2f). However, the number of dead cells per hA is overall still very small (≈1% or less of the total number of cells within an hA). Printing at 4 °C could increase the cell viability window to facilitate longer prints for larger scale bioprinting. Taken together, we show that hA bioinks have suitable properties for extrusion bioprinting into natural polymer matrices as they remain densely packed and highly viable post‐printing. Furthermore, hAs uniformly expressed pluripotency markers NANOG and OCT4 throughout the printed construct 2–4 h after bioprinting (Figure 5d).

Given that downstream therapeutic applications of 3D bioprinted hAs require differentiation into more specialized tissues, we next aimed to validate that the bioprinted hA bioinks are able to differentiate into mature cell types. We applied an adapted version of the cortical organoid differentiation protocol to bioprinted hAs. These differentiated tissues expressed PAX6, a transcription factor, and Nestin, an intermediate filament protein, both of which are expressed in neuroepithelium (Figure 5e). We also applied an adapted vascular organoid differentiation protocol^[^ ^40^ ^]^ on bioprinted pluripotent hAs directly after printing. By day 10 of differentiation, organoids formed a VE‐cadherin+ endothelial cell network containing lumen‐like structures (Figure 5f,g). In both neuroepithelial and vascular differentiations, many of the hAs fused together by day 10 of the differentiation, with the collagen‐Matrigel scaffold remaining intact (Figure 5e,f; Figure S9, Supporting Information). The extent of fusion is dependent on the diffusion in the initial 5–10 min after bioprinting. With lower printing fidelity (associated with bioprinting hA bioinks into insufficiently solidified collagen‐Matrigel matrices), the distance between individual aggregates hinders fusion into contiguous tissue. hAs further from the initially printed structure remain granular as distinct aggregates (Figure 5e,f). Crucially, we observed a high degree of tissue‐specific differentiation at all depths (top, middle, and bottom) of the printed constructs (Figure S9, Supporting Information). Flow cytometry analysis of the bioprinted constructs differentiated into neuroepithelial cells showed that 57.4 ± 10% of SCVI‐15 and 59.0 ± 9.3% of WTC‐11 cells expressed Nestin (Figure 5h) at day 10. Furthermore, 36.5 ± 16% of bioprinted SCVI‐15 and 41.3 ± 10% of bioprinted WTC‐11 cells differentiated into vascular cells expressed VE‐cadherin at day 10 (Figure 5i). Notably, the mean differentiation efficiencies of the bioprinted constructs were lower (neuroepithelium) or higher (vascular) compared to the corresponding differentiations of hAs in suspension. This is likely because hAs are embedded into the collagen‐Matrigel at different time points: for the bioprinted samples, hAs were embedded before initiating differentiation whereas suspension hAs are embedded on day 3 (neuroepithelium) or 5 (vascular) of organoid differentiation. Further optimization of the differentiation protocols can be used to maximize the differentiation into the cell types of interest.

Taken together, we show that bioprinted hAs were able to differentiate into specific lineages, which demonstrates the applicability of the hA bioinks as starting material for producing mature 3D bioprinted tissues. Overall, the automated bioreactor culture of hAs could be achieved at the 250 mL and 1 L scales to produce 1–4 billion cells, and that these hAs can be condensed into a bioink, bioprinted, and differentiated for the production of specialized 3D tissues.

## 3 Discussion

Here, we developed an efficient pipeline for growing hAs at the 250 mL and 1 L scales, bioprinting, and differentiating printed constructs into cell types of interest. Using a data‐driven DoE, we identified optimal growth conditions to culture hAs with monodisperse diameters that could be used for serial passaging or end‐point bioprinting and differentiation. Furthermore, we demonstrated that hiPSCs cultured for three passages in the bioreactors retained key characteristics including the having a desired hA diameter and circularity distribution, and high cell density, viability, and pluripotency marker expression. We also demonstrated that the parameters used in the Ambr 250 modular system were scalable to the BioStat B‐DCU 1 L system, which also yielded hAs with desirable diameters and circularity, cell density and viability, and pluripotency marker expression. After culturing in bioreactors, WTC‐11 cells were karyotypically normal, but a proportion of SCVI‐15 cells contained 1q duplications. Given that 1q duplications are a known recurrent feature in hiPSC cultures which are associated with a proliferative advantage,^[^ ^35^ ^]^ we hypothesize that low levels of cells containing 1q duplications were present in the SCVI‐15 hiPSC stock we obtained. These karyotypically abnormal cells may have preferentially expanded when cultured over many passages. These findings reiterate the importance of keeping stock hiPSC passage numbers low and the need to perform hiPSC line‐specific quality control to identify karyotypic abnormalities.

Functionally, hAs from both 250 mL and 1 L culture systems could differentiate into organoids of ectodermal, mesodermal, and endodermal origin. The hAs were also compacted into a wholly‐cellular, scaffold‐free bioink, which had a suitable yield stress for extrusion bioprinting. Finally, these hA bioinks were bioprinted into a desired shape and differentiated into neuronal and vascular tissues. Our results demonstrate the utility of hAs cultured in large scale for key downstream applications in tissue engineering, such as customized cell type‐specific spatial architecture and represent a direct, streamlined hiPSC‐to‐bioprinted tissue process.

This study demonstrates a method for establishing a continuous expansion and differentiation pipelines in bioreactors to fuel future larger scale bioprinting efforts. For long‐term expansion, hAs may undergo multiple passages to sustain the logistics for such applications. We recommend that the quality checks conducted in this study, such as verifying karyotype, pluripotency marker expression, viable cell density, and distribution of hA size and circularity, be routinely conducted to ensure the highest standards in such cultures. Furthermore, it may be beneficial in future studies to differentiate hAs into mature tissue aggregates inside the bioreactor, that is, prior to bioprinting.

Bioprinting hAs or organoid‐based bioinks as opposed to printing single cell bioinks could drastically reduce the print times for manufacturing larger tissues.^[^ ^9^ ^]^ Bioinks composed of compacted slurries of hAs instead of slurries of single cells also exhibit higher yield stresses, enabling the printing of more mechanically stable constructs. Furthermore, bioprinting hAs also streamlines the manufacturing pipeline because it omits a time‐ and resource‐demanding, and potentially cell injurious dissociation step in between harvesting and printing the bioink. While studies directly comparing the viability and functional phenotypes of printed hA bioinks and single cell hiPSC bioinks have not been done, a previous study showed that liver constructs assembled from biophrinted hepatic spheroids had more favorable viability, hepatic marker expression, and metabolic phenotypes compared to tissues bioprinted with bioinks comprised of single hepatic cells.^[^ ^53^ ^]^ This finding suggests that bioprinting cellular aggregates may improve the viability and biological phenotypes of the assembled construct. Finally, using autologous wholly‐cellular bioinks may bypass the need to complete additional regulatory quality checks associated with the use of non‐autologous materials for transplantation therapy, which would further streamline the manufacturing pipeline.^[^ ^54^ ^]^

In future applications for bioprinting larger constructs, it would be useful to characterize microstructures, macro‐architectures, and tissue‐specific cell densities of the constructs to further verify that these constructs better recapitulate tissue function. Since larger and thicker bioprinted constructs require perfusion of tissues via vasculature for survival, sufficient coverage of perfusable interconnected vascular networks across the tissues should be verified. Further scale up of bioink production for whole organ‐scale biofabrication will undoubtedly raise the issue of extended print times and subsequent poor cell viability. Previous approaches to parallelize multimaterial printing could offer a way to reduce the print time of larger quantities of wholly cellular bioinks.^[^ ^55^ ^]^ Furthermore, while our bioprinted structures are quasi‐planar in nature, embedded 3D bioprinting in a deeper bath of collagen‐Matrigel could enable more complex structures to be formed. Alternatively, a xeno‐free and wholly‐cellular bioprinting scaffold could be envisioned in the future if a sacrificial support bath, such as microparticles of gelatin (i.e., FRESH)^[^ ^4^ ^]^, was used to support the wholly cellular bioink. In this method, bioprinted constructs are held together by fibrin, the cleaved form of fibrinogen which may be acquired in an autologous blood draw.^[^ ^56^ ^]^

In conclusion, our work demonstrates the development of a pipeline to produce scalable quantities of densely‐cellular bioinks for 3D bioprinted tissues. We produced 1 billion cells in 5‐day cultures at a 250 mL scale and 4 billion cells in 4‐day cultures at a 1 L scale. For future work aiming to manufacture larger solid organs at the native density of 100–200 million cells mL^−1^,^[^ ^5^, ^9^, ^57^ ^]^ cells in the order of billions will be required.^[^ ^2^ ^]^ Our work shows the feasibility of printing approximately 1 cm × 1 cm × 0.1 cm tissues, and we project the need to scale bioreactor volumes to at least 10 L to produce sufficient cells to manufacture therapeutic‐scale organ tissues with tens of billions of cells.^[^ ^58^ ^]^ In the future, organ biofabrication strategies will undoubtedly require thousands of experimental iterations to optimize tissue function, and will need efficient pipelines for generating billions, or even trillions, of cells.

## 4 Experimental Section

### Adherent Culture of hiPSCs

SCVI‐15 human induced pluripotent stem cells (Stanford Cardiovascular Institute Biobank) and WTC‐11 human induced pluripotent stem cells (Gladstone Institutes) were cultured in NutriStem hPSC XF media (Sartorius 05‐100‐1A) on 12 µg cm^−2^ GelTrex (hESC‐qualified) (Thermo Fisher Scientific A1413302). For passaging, hiPSCs were washed with PBS without calcium and magnesium, and incubated in Gentle Cell dissociation reagent (PBS without calcium and magnesium (Corning 21‐049‐CV), 0.5 mm EDTA (Sigma EDS‐500G), 1.8 g L^−1^ sodium chloride (Sigma S7653)) for 10 min at 37 °C, resuspended in media in a 1:1 volume with Gentle Cell, centrifuged for 300 × *g* for 5 min, and seeded at 20 000 cells cm^−2^ (SCVI‐15) or 26 666 cells cm^−2^ (WTC‐11) with 10 µm Y‐27632 dihydrochloride (BioGems 21293823). Media was refreshed daily. Cells were routinely tested for mycoplasma and were karyotyped. The SCVI‐15 line is a well‐characterized cell line used by researchers at the Stanford Cardiovascular Institute (SCVI) and available through the SCVI biobank. The WTC‐11 line was also well‐characterized by the Allen Institute and other researchers, was widely used in literature (over 50 citations^[^ ^59^ ^]^), and available through the Coriell Institute.

### hiPSC Suspension Culture in Ambr 250 Modular

hAs were cultured using the Ambr 250 modular automated bioreactor system (Sartorius 001–8A64) in unbaffled vessels fitted with a single elephant ear impeller (Sartorius 001–2A33). hiPSCs were seeded on day 0 as single cells in 240 mL NutriStem hPSC XF media supplemented with 40 ng mL^−1^ bFGF (Peprotech 100–18B) and 10 µm Y‐27632 dihydrochloride. A 10 mL buffer volume was used to allow for base addition to the culture and to avoid liquid overflow in the bioreactor. In select conditions screened, cells were cultured in media supplemented with 4 mg mL^−1^ polyvinyl alcohol (PVA, Sigma P8136) solution (Table S2, Supporting Information). PVA was included as a variable in the initial design space of the DoE. However, subsequent multivariate data analyses indicated that PVA had a negligible effect on the desired output parameters, and in light of this, PVA was excluded in the subsequent experiments. pH was controlled at pH 7.3 ± 0.2, which was the optimum pH for cell culture and maintaining media components in solution. pH control was regulated by a real‐time feedback loop initiated by measurements from the internal pH probe, and responses by addition of 1 m sodium bicarbonate (VWR 470302–440) or CO_2_ gas input. The DO setpoint was set at the equilibrium partial pressure obtained when cell‐free media was stirred at 37 °C with 5.25% oxygen in the headspace.

Due to the small hA sizes, media changes on day 1 were done by centrifuging the culture at 300 × *g* for 7 min to pellet hAs. hAs were resuspended in fresh media. On subsequent days, media changes were done by allowing the hAs to gravity‐settle in the vessel for 10–20 min, and refreshing 80% of the media.

For sampling, the impeller was manually rotated and a 10 mL serological pipette was used to triturate the suspension to suspend and distribute hAs in the media.

For dissociation into single cells, hAs were washed with PBS without calcium and magnesium, and incubated in Accutase (Innovative Cell Technologies AT‐104) for 5–7 min stationary at room temperature. Cells were pelleted and resuspended in culture medium. For serial passaging, cells were passed through a 40 µm filter, and reseeded into a new vessel. Cells were counted using a Countess 3 FL Automated Cell Counter (Thermo Fisher Scientific). Cell density measurements were corrected to account for cumulatively sampled volumes.

### hiPSC Suspension Culture in BioStat B‐DCU

hAs were cultured using the BioStat B‐DCU twofold Flexible 120V (Sartorius) and UniVessel Glass CC 2 L SW automated bioreactor system (Sartorius, UNIVESSELMU) in 1 L of medium.

hiPSCs were seeded on day 0 as single cells in 1 L NutriStem hPSC XF media supplemented with 40 ng mL^−1^ bFGF and 10 µm Y‐27632 dihydrochloride. pH was controlled at pH 7.3 ± 0.2, which was the optimum pH for cell culture and maintaining media components in solution. pH control was regulated by a real‐time feedback loop initiated by measurements from the internal pH probe, and responses by addition of 1 m sodium bicarbonate or CO_2_ gas input. The DO setpoint was set at the equilibrium partial pressure obtained when cell‐free media was stirred at 113 RPM at 37 °C with 5.25% oxygen in the headspace. From day 1 of the culture, perfusion medium exchange was started. Medium was extracted via a dip tube with a stainless steel 20 µm pore head at the rate of one vessel volume per day (VVD), and fresh NutriStem media supplemented with 40 ng mL^−1^ bFGF was pumped in via another dip tube under a weight control loop.

Samples were taken via a sampling line during suspension culture. Dissociation and cell counting were performed as described in Section 4.2, except 100 U mL^−1^ DNaseI (Worthington LS002139) was supplemented during incubation with Accutase, and incubation was 30–60 min at 37 °C on an orbital shaker.

### hA Size and Circularity Analysis

Brightfield images were imported into MATLAB R2021A, and a custom script based on Chao‐Yen Yuh's nd2read^[^ ^60^ ^]^ was used to convert the ND2 files into one‐channel 8 bit depth PNG images. The images were then converted to black and white using a global αthreshold ranging from 0.4 to 0.6. Segmentation was done via an algorithmic Rough Hough Transform. Each pixel was taken as a potential center of circle (*A, B*), and the circle equation:

(2) $${\left(x-A\right)}^{2}+{\left(y-B\right)}^{2}=R^{2}$$

was solved for a range of pixel radius values. Once the pixel values were solved, if the center pixel *(A, B)* and the solved pixel value (*x_i_*, *y_i_*) were also black, a positive vote was accumulated for that center (*A, B*) at that particular radius *R*. By virtue of the most votes, a center (*A, B*) and a radius *R* were chosen as the size and location of the hA. For split votes, a non‐negative hyperparameter, sensitivity, ranging from 0–1 was used to algorithmically select the larger *R* if the sensitivity was closer to 1. In order to detect as many hAs per image as possible, and keeping computation times and errors to a minimum, each picture was run through this segmentation algorithm five times, each with a different range of pixel radii to search for features between 20–200, 40–200, 60–200, 80–200, and 90–200 pixels, as well as different sensitivity values (0.72, 0.76, 0.80, 0.88, 0.92). Any repeat hA identification between the algorithm runs were disregarded, and a fully segmented image was taken by stacking all of the segmentations per run.

Circularity is defined as a ratio of the area of a circle to its perimeter, with a perfect circle having a circularity of 1. Circularity is defined as:

(3) $$\mathrm{Circularity}=\frac{4\pi A}{P^{2}}$$

where *A* = area, *P* = perimeter.

The area of each hA was calculated by counting the number of black pixel dots within the segmentation region. In order to account for irregularities in hA shape, the area of each segmented region was calculated by taking the harmonic mean of the area of the segmented region if the radius was 4 pixels smaller, and 4 pixels larger than the segmented radius found by the rough Hough transform. The circularity was then found by dividing the harmonic mean by the area of a circle with the radius of the segmented hA.

For all data visualization and plots, custom scripts were written (permanently recorded script^[^ ^61^ ^]^; up‐to‐date script^[^ ^62^ ^]^). All computation and analysis were carried out with MATLAB R2021A.

### Flow Cytometry Analysis

hAs were dissociated using Accutase as described above. hA‐derived cardiac aggregates were dissociated using Collagenase IV (Gibco 17‐104‐019) for 25 min and TrypLE (Gibco 12–605) for 10 min. Neuroepithelial aggregates were dissociated in TrypLE supplemented with 100 U mL^−1^ DNaseI (Worthington LS002139) for 15 min. Bioprinted constructs differentiated into neuroepithelium or vascular cells, and vascular organoids were dissociated using a cocktail consisting of 3 U mL^−1^ Dispase (STEMCELL Technologies 07913), 2 U mL^−1^ Liberase (Roche 05 401 119 001), and 100 U mL^−1^ DNaseI for 25–40 min, followed by TrypLE supplemented with 100 U mL^−1^ DNaseI for 10 min. Definitive endoderm cells were dissociated using Accutase for 5 min. 200 000 or 500 000 cells were aliquoted into a 96‐well plate. Cells were stained with a live/dead marker for 30 min on ice, washed twice with 0.1% bovine serum albumin (BSA) (Sigma A3311) in PBS. Samples with Fc receptors were incubated with Fc blocker (BioLegend 422302) for 10 min at room temperature. Surface marker antibodies (Table S4, Supporting Information) were diluted in 0.1% BSA in PBS and incubated for 30 min on ice. Samples were washed thrice with 0.1% BSA, fixed with BD CytoFix (BD 554714) for 15 min and washed thrice in 0.1% BSA. Samples were permeabilized and blocked with 1× BD Perm/Wash (BD 554723) for 15 min. Antibodies against intracellular markers (Table S4, Supporting Information) were diluted in Perm/Wash and incubated for 45 min on ice. Samples were washed thrice with Perm/Wash and resuspended in 0.1% BSA for analysis. For all buffer exchanges, samples were pelleted at 500 × *g* for 5 min at 4 °C and decanted. Samples were analyzed on a NovoCyte Quanteon Flow Cytometer at the Stanford Shared FACS Facility. Color compensation and controls were performed using single color and isotype controls. Example gating strategies are shown in Figure S11, Supporting Information. Antibodies and dilutions are listed in Table S4, Supporting Information. Data was analyzed using FlowJo v. 10.8.1.

### Design of Experiments Determination and Multivariate Data Analysis

The experimental conditions were generated, and the results analyzed by MODDE, a Design of Experiments (DOE) program, to determine which experimental factors affected the responses of interest. The factors explored were initial speed, final speed, and seeding density (Table S1, Supporting Information).  MODDE Pro Version 13.0.2.34314 from Sartorius Stedim Data Analytics AB was utilized for the creation of the design and subsequent analysis with standard settings, except for the use of PLS (Partial Least Squares) due to the irregular design space. The factor settings and responses analyzed are found in Tables S1–S3 and Figure S4, Supporting Information.

### Rheological Characterization of hAs

The rheology of the compacted hAs was measured on a commercial stress‐controlled rheometer (TA Instruments AR‐G2) with a thermoelectric Peltier plate for temperature control (±0.01 °C). Slurries of SCVI‐15 hAs, WTC‐11 hAs, or SCVI‐15 or WTC‐11 single cells, were compacted into 1 mL syringes (Hamilton Series 1000 Gastight Syringe), and 50 µL of the sample was directly extruded onto the rheometer plate preset at 20 °C. Both the plate and disc (8 mm in diameter) were coated with sandpaper (grit number 320) to prevent wall slip, the gap height was set to 1 mm. To measure the linear viscoelastic response, an oscillatory shear strain was applied over a frequency range 0.1–10 rad s^−1^ at an amplitude of 1%. To measure the amplitude response, the frequency was set at 0.5 Hz and the oscillatory amplitude was varied from10^−5^ to 0.5. The steady‐shear response was measured by sweeping the constant shear rate from 5 to 0.01 s^−1^ to eliminate transient responses, followed by an identical protocol with the shear rate increasing to ensure the absence of data hysteresis. To fit the viscoelastic responses of these slurries, a five‐parameter fractional Jeffrey model (FJM) was used, which can be represented by a four‐parameter fractional Maxwell model connected with a dashpot (Newtonian) component in parallel. Using the Caputo form for the fractional derivative,^[^ ^63^ ^]^ the storage and loss moduli can be expressed^[^ ^64^ ^]^ as:

(4) $$G^{\prime }\left(\omega \right)=\frac{\left(V\omega^{\alpha }\right)\;{\left(G\omega^{\beta }\right)}^{2}\cos\left(\pi \alpha /2\right)+{\left(V\omega^{\alpha }\right)}^{2}\left(G\omega^{\beta }\right)\cos\;\left(\pi \beta /2\right)\;}{{\left(V\omega^{\alpha }\right)}^{2}+{\left(G\omega^{\beta }\right)}^{2}+2VG\omega^{\alpha +\beta }\cos\left[\pi \frac{\left(\alpha -\beta \right)}{2}\right]}$$

(5) $$\begin{array}{c} G^{\prime \prime }\left(\omega \right)=\frac{\left(V\omega^{\alpha }\right){\left(G\omega^{\beta }\right)}^{2}\sin\left(\pi \alpha /2\right)+{\left(V\omega^{\alpha }\right)}^{2}\left(G\omega^{\beta }\right)\sin\left(\pi \beta /2\right)}{{\left(V\omega^{\alpha }\right)}^{2}+{\left(G\omega^{\beta }\right)}^{2}+2\mathrm{VG}\omega^{\alpha +\beta }\cos\left[\pi \left(\alpha -\beta \right)/2\right]}+\eta_{0}\omega \end{array}$$

### hA Printing

To prepare the hA bioink, hAs were filtered through a 400 µm cell strainer (PluriSelect). The filter was rinsed three times with DMEM/F12 (Thermo Fisher Scientific 11320), and hAs were allowed to settle. The supernatant was aspirated, hAs were resuspended in 10 mL DMEM/F12, and centrifuged at 100 × *g* for 2 min at 4 °C. The cells were then resuspended in 300 µL DMEM/F12, and then transferred to a 1 mL syringe (BD 309628). The syringe was centrifuged at 4 °C, 300 × *g* for 3 min to compact hAs, and the supernatant was aspirated. hAs could be left on ice for up to three hours prior to printing. The shortest duration between compaction and bioprinting was ≈15 min due to the following preparations and time taken to remove supernatant, insert the plunger, and affix the syringe onto the bioprinter. The compacted hAs in the syringe affixed to the bioprinter were kept at 20 °C. A tapered nozzle (Nordson 7005009) with 0.58 mm inner diameter was used to print the hAs into a basement gel cast into a 35 mm dish or 6‐well plate. The basement gel was prepared by mixing rat tail collagen (Corning 354249 or Advanced Biomatrix 5153) (final concentration 4 mg mL^−1^) and Matrigel (Corning 354230) (final concentration 25% v/v) into DMEM/F12. 10× PBS (MP Biomedicals 281030) was supplemented at 10% collagen volume, and 1 m NaOH was supplemented at 2.3% collagen volume. All reagents were kept on ice, and pipette tips were pre‐cooled by pipetting cold PBS briefly before handling collagen and Matrigel. 1 mL of the basement gel was cast into each well and incubated at 37 °C for up to 8 min to allow partial gelling before printing in a biosafety cabinet. A custom‐built, rectilinear extrusion 3D bioprinter was used for printing. The custom bioprinter employed three linear actuators (*X*‐, *Y*‐, and *Z*‐axes) and one custom syringe pump for extrusion. Motion control was achieved using a Rumba control board loaded with open‐source Marlin firmware for computer numerical control (CNC) machining or 3D printing. After printing, the wells were incubated for up to 8 more minutes at 37 °C until fully gelled before DMEM/F12 with penicillin‐streptomycin (Thermo Fisher Scientific 15140122) was added to submerge the cells. The printed constructs were immediately differentiated into cortical neurons or incubated for 1 day before initiating differentiation into vascular endothelial cells.

Controls for live/dead stain were printed either through the 0.58 mm diameter nozzle or manually extruded from the syringe without a nozzle to simulate negligible shear stress. Live/dead (Thermo Fisher Scientific L3224) stain was added to the basement gel and media before casting and printing. The tissues were imaged using an epifluorescence microscope after a 10–30 min incubation in the live/dead stain at 37 °C.

### Cortical Organoid Differentiation

Cortical organoid differentiation was performed for 45 days as previously described^[^ ^36^ ^]^ based on adaptations of established protocols,^[^ ^37^, ^38^, ^39^ ^]^ using hAs collected on day 3 from the bioreactor culture. Briefly, hAs were cultured in neural induction medium (NIM), then embedded in collagen/Matrigel droplets and cultured in NIM. Subsequently, step‐wise neural differentiation was achieved by transitioning organoids from neural differentiation media (NDM) 1, to NDM2, then NDM3. The small molecules and growth factors used included 5 µm SB431542 (Biogems 3014193), 100 nm LDN193189 (Biogems 1066208), 10 ng mL^−1^ VEGF (PeproTech 100–20), 20 ng mL^−1^ EGF (R&D 236‐EG), 20 ng mL^−1^ FGF2 (PeproTech 100–18B), and 20 ng mL^−1^ BDNF (PeproTech 450‐02). Day 45 organoids were fixed with 4% paraformaldehyde (Electron Microscopy Systems 15710). Printed hAs were differentiated using a similar protocol, but received daily media changes, following the same timeline as for free‐floating hAs.The collagen‐Matrigel matrices were intact up to day 10 with no apparent disintegration.

### Vascular Endothelial Cell Differentiation

Vascular endothelial cell differentiation was performed as previously described^[^ ^40^ ^]^ using hAs collected on day 3 from the bioreactor culture. Briefly, hAs were incubated with 12 µm CHIR and 30 ng mL^−1^ BMP‐4 for 3 days, followed by 100 ng mL^−1^ VEGF and 2 µm forskolin for two days. On day 5 of the differentiation, hAs were subsequently embedded in a collagen‐Matrigel matrix, and incubated in media containing 100 ng mL^−1^ VEGF, 100 ng mL^−1^ FGF, and 15% FBS (Cytiva SH30070.01). Printed hAs were differentiated using a similar protocol above, but were not embedded into a collagen‐matrix on day 5 of the differentiation. The collagen‐Matrigel matrices were intact up to day 10 with no apparent disintegration.

### Cardiomyocyte Differentiation

hAs collected on day 3 or 4 of culture were transferred to a T‐25 flask passivated with 0.1% pluronic (Sigma P2443) and grown, with daily media changes, on a shaker set at 52 RPM until diameters reached 250–300 µm. Differentiation to cardiomyocytes was achieved via temporal Wnt modulation, according to previously published protocols.^[^ ^5^, ^65^, ^66^ ^]^ Briefly, hAs were treated with 4 µm Gsk3 inhibitor CHIR99021 (BioGems 2520691) for two days followed by 4 µm Wnt inhibitor iWR1 (BioGems 1128234) for three days.

### Intestinal Organoid Differentiation

hAs were differentiated into intestinal tissue as previously described.^[^ ^42^ ^]^ Briefly, hAs collected from the bioreactor on day 3 were dissociated, replated, and grown until colonies reached 50–60% confluency. Endoderm differentiation was initiated by incubating in 100 ng mL^−1^ Activin A (R&D Systems 338‐AC) and 2 mm l‐glutamine for 1 day, followed by a daily media change with 100 ng mL^−1^ Activin A, 2 mm l‐glutamine, and 0.2% v/v FBS (Cytiva SH30070.01) for 2 days. Cells were then differentiated into mid‐ and hind‐gut by culturing in medium containing 500 ng mL^−1^ WNT3A (R&D Systems 5036‐WN), 500 ng mL^−1^ FGF4 (R&D Systems 235‐F4), and 2 mm l‐glutamine for 3 to 4 days, with daily media refresh. Budding spheroids were harvested by rinsing wells with culture media and were embedded in Matrigel for maturation. Once embedded, organoids were fed every four days or until media turns yellow with intestine growth medium, which contained 500 ng mL^−1^ Rspondin1 (R&D Systems 4645‐RS), 100 ng mL^−1^ noggin (R&D Systems 6057‐NG), and 100 ng mL^−1^ EGF (Peprotech AF‐100‐15).

### Wholemount Immunofluorescence

Tissues were fixed at room temperature with 4% paraformaldehyde. Duration of fixation was optimized according to the depth of the tissues. Adherent tissues were fixed for 10 min; hAs, embedded aggregates or organoids, and bioprinted tissues were fixed for 20 min, and day 45 cortical organoids were fixed for 30 min. Fixed organoids were permeabilized with 0.1% (v/v) Triton‐X (Millipore Sigma 1.12298.0101) in 1x animal‐free blocking buffer (AFBB, Vector Laboratories SP‐5030‐250) for 30 min, then washed twice with AFBB. Primary antibodies were diluted in AFBB and incubated for 16–24 h at 4 °C with gentle agitation (Table S5, Supporting Information). Samples were washed three times with 0.1% (v/v) Tween‐20 (Millipore Sigma 655206) and incubated in secondary antibodies for 16–24 h at 4 °C with gentle agitation. Samples were washed three times with 0.1% Tween‐20, incubated in DAPI (Millipore Sigma #D9542) or Hoechst (Apex Bio, Cat #A3472) for 20 min at room temperature, and washed three times. Organoids were cleared with EasyIndex (LifeCanvas Technologies) to render them optically transparent, and mounted on a coverglass for imaging.

### Cryosectioning and Immunofluorescence

Fixed organoids were incubated in 30% wt/vol sucrose in PBS with calcium and magnesium for 2 days at 4 °C. Samples were transferred and incubated in a 1:1 solution of 30% wt/vol sucrose in PBS with calcium and magnesium (Corning 21‐030‐CV) and Optimal Cutting Temperature compound (OCT) (Tissue‐Tek 4583) for 90 min. Samples were placed in mold and frozen in 100% OCT solution at −20 °C on a Peltier cooler. Samples were sectioned into 40 µm slices and transferred onto a Superfrost Plus glass slide (VWR 48311–703). Sections were stored at −20 °C before immunostaining. On the day of immunostaining, samples are thawed at room temperature for 10 min and rehydrated in AFBB for 10 min. The staining protocol was as described in the wholemount immunofluorescence methods. Samples were mounted using Vectashield HardSet Antifade Mounting Medium (Vector Laboratories H‐1400‐10).

### Brightfield Imaging

Brightfield and widefield images were acquired using a Nikon Eclipse Ts‐2R inverted microscope and a 4× Plan Fluor 0.13 NA or 10× Plan Fluor 0.30 NA objective lens and a Nikon DS‐Qi2 CMOS camera. NIS‐Elements BR 5.21.01 was used to acquire images.

### Confocal Imaging and Image Analysis

Stained vascular, cardiomyocyte, and cortical organoids were imaged using a Leica SP8 DMI 6000 inverted confocal microscope through a 20× HC PL APO 0.75 NA Corr CS2 oil immersion or 40× HC PL APO CS2 1.3 NA oil immersion objective. The microscope is equipped with three Hybrid‐GaAsP detectors and two standard fluorescent photomultiplier tubes. Leica LAS X Premium software was used to acquire images. Stained hAs and intestinal organoids were imaged using an Olympus IX81F‐3 fluorescence motorized microscope combined with a confocal laser scanning FV1000 FLUOVIEW system, through a 30× UPlanSApo 1.05 NA oil immersion objective. Olympus Fluoview Ver 4.2 software was used to acquire images. Composite image generation and 3D rendering were performed using FIJI and ImageJ bundled with 64‐bit Java 1.8.0_172.^[^ ^67^, ^68^, ^69^ ^]^ Image stitching was performed using the Pairwise Stitching plugin.^[^ ^69^ ^]^

### Statistical Analysis

Data was not pre‐processed prior to analysis. Plots either show all data points and were annotated with the mean and standard deviation (normal data) or median and interquartile range (non‐normal data), as specified in the figure legends. Data in all figures were aggregated from 2–4 experimental replicates, with specific replicate numbers stated in figure legends. Where appropriate, unpaired two‐tailed *t*‐tests were used to assess whether differences in two population means were statistically significant. For non‐normal data sets (e.g., hA diameters in Figures 1 and 2), the non‐parametric Mann‐Whitney U‐test was used to assess differences in the distributions of two populations. Instances of when statistical tests are used are stated in figure legends. ns: not significant (*p* > 0.05), *p < 0.05, **p < 0.01, **** p<0.0001. Data was plotted and analyzed using GraphPad Prism v.9.3.1 or Matlab R2020B software.

## Conflict of Interest

M.A.S.‐S. owns stock in Formlabs Inc., a 3D printing company, is a consultant for 3D Systems, a 3D printing company, and serves on the scientific advisory board of Acoustica Bio, a materials formulation company, and Mooji Meats, a synthetic meat company. The Ambr 250 modular and BioStat B‐DCU bioreactor systems were provided on loan from Sartorius Stedim; NutriStemhPSC XF medium was supplied by Sartorius Stedim. A.H., S.D., D.T., P.T., D.S., R.L, N,D., K.K., and Q.V. are employees of Sartorius Stedim, which is the manufacturer of the Ambr 250 modular and BioStat B‐DCU bioreactors and NutriStemhPSC XF medium.

## Author Contributions

M.A.S.‐S. conceived the study. D.L.L.H., S.L., and M.A.S.‐S. designed the experiments and supervised the work. D.L.L.H. and J.E.H. carried out hA cultures, daily measurements of cell density, viability, and glucose concentrations. D.L.L.H. acquired hA 2D projections. D.L.L.H., S.L., J.D.W., and J.E.H. contributed to the maintenance and set‐up of the BioStat B‐DCU bioreactor system. D.L.L.H., S.L., S.D., D.T., P.T., D.S., and M.A.S.‐S. contributed to the optimization of culture conditions and bioreactor programming. S.S. wrote the image analysis pipeline and analyzed the distribution of hA diameters and sizes, curated the pipeline code and data, and generated the respective graphs. A.H. conducted the MVDA. S.L., D.L.L.H., T.T., and T.K.T. contributed to flow cytometry characterization. S.L., D.L.L.H., T.T., J.D.W., D.K., and L.G.F. contributed to immunofluorescence staining, cryosectioning, and confocal image acquisition. S.L., D.K., H.T.L., T.T., and D.L.L.H. contributed to organoid differentiations. J.D. and M.H. acquired rheological measurements, and J.D. carried out the respective analysis. J.D.W. and D.K. carried out the 3D bioprinting of hAs. J.D.W. and S.L. carried out the viability assay. D.L.L.H., S.L., J.D., and M.A.S.‐S. wrote and edited the manuscript. L.G.F. and T.K.T. proofread the manuscript. K.K., Q.V., R.L., N.D., and M.A.S.‐S were involved in project administration and funding and resource acquisition.

## Supporting information

Supporting Information

Supplemental Movie 1

Supplemental Movie 2

Supplemental Movie 3

## Data Availability

The data that support the findings of this study are available from the corresponding author upon reasonable request.
