# Supplementary material for: Large‐Scale Production of Wholly Cellular Bioinks via the Optimization of Human Induced Pluripotent Stem Cell Aggregate Culture in Automated Bioreactors
Source: Adv Healthc Mater. 2022 Nov 22;11(24):2201138. doi: 10.1002/adhm.202201138 (PMC10234214; doi:10.1002/adhm.202201138)
Supplement: Supplementary file 1 — Supporting Information [file ADHM-11-2201138-s003.pdf]

ADVANCED HEALTHCARE MATERIALS

Supporting Information

for *Adv. Healthcare Mater.*, DOI 10.1002/adhm.202201138

Large-Scale Production of Wholly Cellular Bioinks via the Optimization of Human Induced Pluripotent Stem Cell Aggregate Culture in Automated Bioreactors

*Debbie L. L. Ho, Stacey Lee, Jianyi Du, Jonathan D. Weiss, Tony Tam, Soham Sinha, Danielle Klinger, Sean Devine, Art Hamfeldt, Hope T. Leng, Jessica E. Herrmann, Mengdi He, Lee G. Fradkin, Tze Kai Tan, David Standish, Peter Tomasello, Donald Traul, Noushin Dianat, Rukmini Ladi, Quentin Vicard, Kishore Katikireddy and Mark A. Skylar-Scott**

Supporting Information

Large-Scale Production of Wholly Cellular Bioinks via the Optimization of Human Induced Pluripotent Stem Cell Aggregate Culture in Automated Bioreactors

*Debbie L. L. Ho[a], Stacey Lee[a], Jianyi Du[a], Jonathan D. Weiss[a], Tony Tam[a], Soham Sinha[a], Danielle Klinger[a], Sean Devine[b], Art Hamfeldt[b], Hope T. Leng[a], Jessica E. Herrmann[a, c], Mengdi He[d], Lee G. Fradkin[a], Tze Kai Tan[e, f, g], David Standish[b], Peter Tomasello[b], Donald Traul[b], Noushin Dianat[h], Rukmini Ladi[b], Quentin Vicard[h], Kishore Katikireddy[b], Mark A. Skylar-Scott[a, i, j]**

[a] Debbie L. L. Ho, Stacey Lee, Jianyi Du, Jonathan D. Weiss, Tony Tam, Soham Sinha, Danielle Klinger, Hope T. Leng, Jessica E. Herrmann, Lee G. Fradkin, Mark Skylar-Scott
Department of Bioengineering, Stanford University, Stanford, California, USA.
E-mail: skyscott@stanford.edu

[b] Sean Devine, Art Hamfeldt, David Standish, Peter Tomasello, Donald Traul, Rukmini Ladi, Kishore Katikireddy
Sartorius Stedim North America Inc. 565 Johnson Avenue, 11716 Bohemia, New York, USA.

[c] Jessica E. Herrmann
School of Medicine, Stanford University, Stanford, California, USA

[d] Mengdi He
Materials Science and Engineering, Stanford University, Stanford, California, USA.

[e] Tze Kai Tan
Institute of Stem Cell Biology and Regenerative Medicine, Stanford University School of Medicine, Stanford, California, USA.

[f] Tze Kai Tan
Department of Genetics, Stanford University School of Medicine, Stanford, California, USA.

[g] Tze Kai Tan

Department of Pathology, Stanford University School of Medicine, Stanford, California, USA.

[h] Noushin Dianat, Quentin Vicard
Sartorius Stedim France S.A.S., Zone Industrielle les Paluds, Avenue de Jouques CS 71058. 13781 Aubagne Cedex, France.

[i] Mark A. Skylar-Scott
Basic Science and Engineering Initiative, Children's Heart Center, Stanford University, Stanford, California, USA.

[j] Mark A. Skylar-Scott
Chan Zuckerberg Biohub, San Francisco, CA 94158, USA

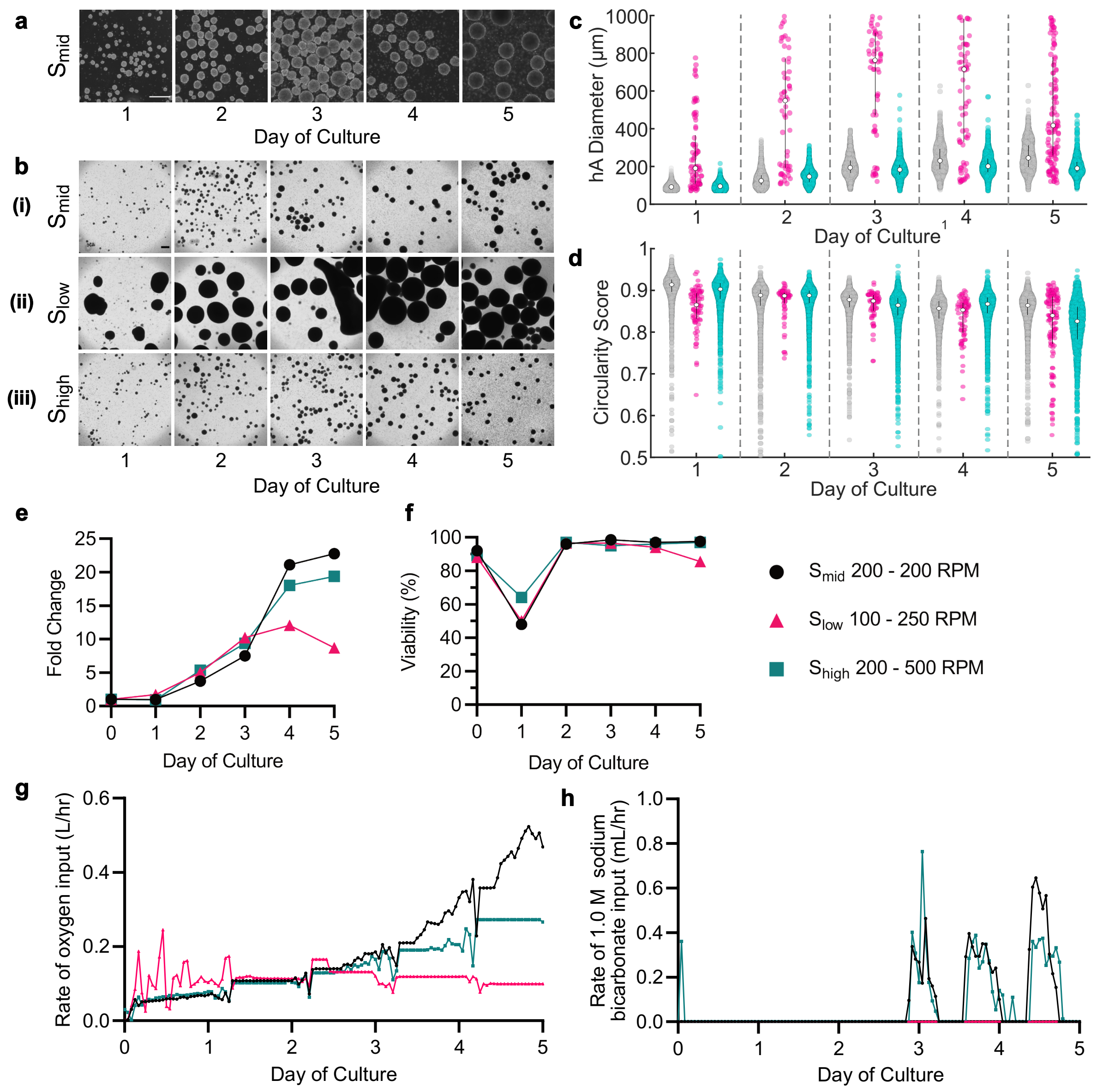

Figure S1. Optimization of impeller speeds to produce SCVI-15 hAs with desired characteristics for 3D bioprinting and differentiation. Three selected experimental conditions from the DoE screen using SCVI-15 hiPSCs are shown. (a) Brightfield images of hAs cultured in the S_{mid} condition from days 1 - 5. (b) Images of hAs over five days of culture at different spin speeds and the resulting hA (c) diameter distribution and (d) circularity distribution. (e) Fold expansion, (f) viability, (g) rate of oxygen input, and (h) rate of base addition (1.0M sodium bicarbonate) for all three conditions are shown. Key: Black/gray, S_{mid}, 200 RPM constant; Pink, S_{low}, 100 - 250 RPM; Teal, S_{high}, 200 - 500 RPM. (a, b) Scale bar, 400µm. (c) Only the S_{low} sample had hAs with diameters >1000 µm, which were excluded from the graph. The total number of data points across day 1 - 5 was 356, and 37 data points were > 1000 µm (10%). (d) 99.5% of hAs were distributed between circularity

scores of 0.5 - 1; data with scores below 0.5 were excluded. (c, d) Median ± IQR shown; n > 200 hAs per condition per day. n = 1 shown for each condition.

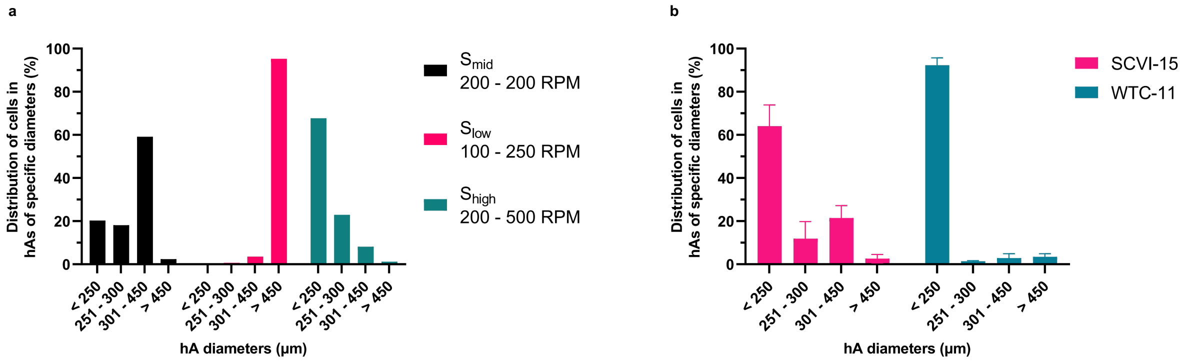

Figure S2. (a) Distribution of hA diameters on day 5 of culture. (b) Distribution of hA diameters in serial passages of SCVI-15 and WTC-11 on day 3 of culture (mean ± s.d.), n=3 replicates per cell line.

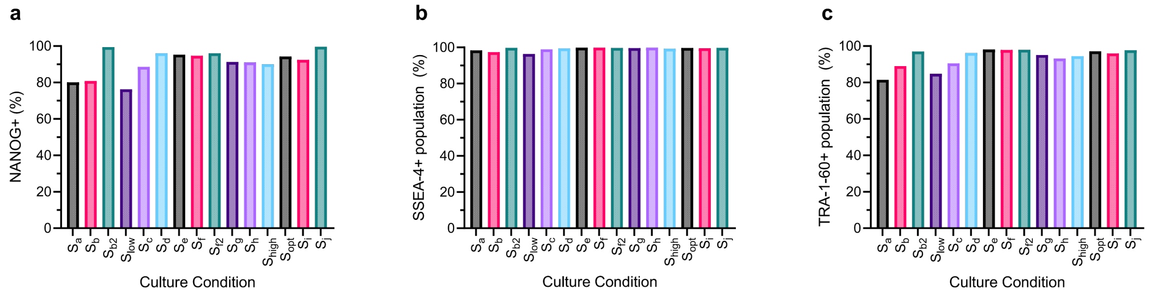

Figure S3. Percentage of the population expressing the pluripotency markers (a) NANOG, (b) SSEA-4 and (c) TRA-1-60 for each set of conditions in the DoE screen. n=1 replicate per condition. Detailed information for each DoE condition is supplied in Table S1.

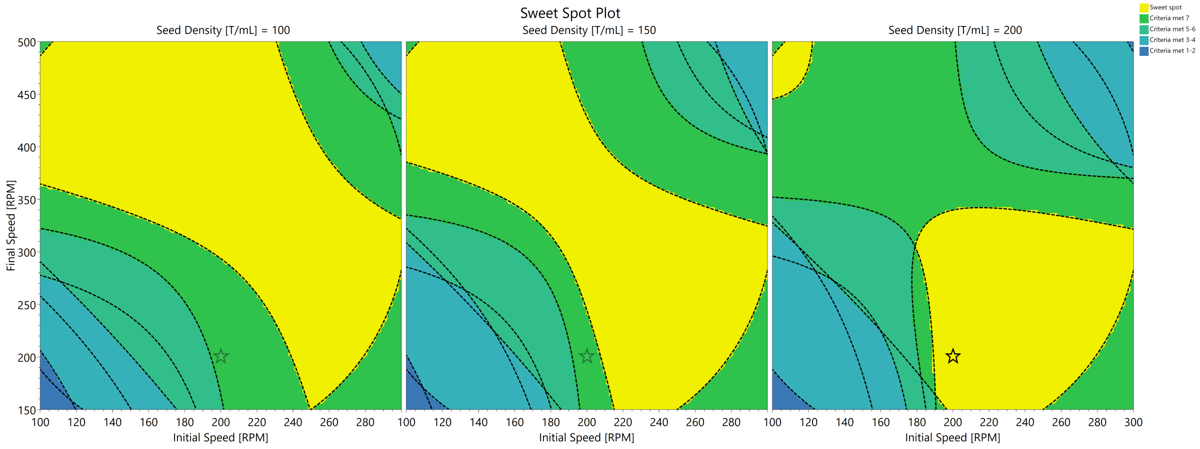

Figure S4. Sweet spot plot for predicting the optimal culture conditions. Yellow regions indicate where the required responses are met with the factor settings outlined in Table S2.

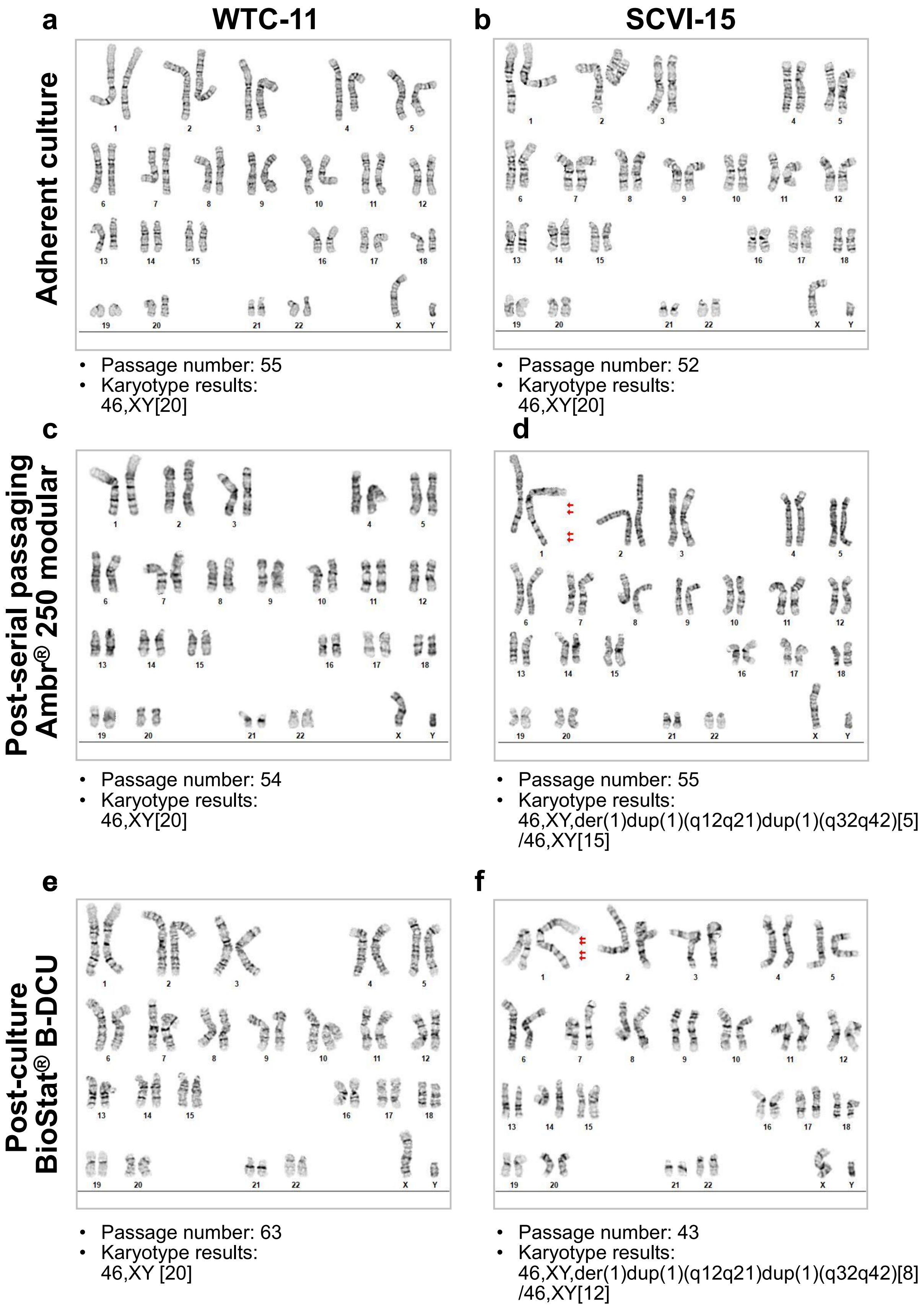

Figure S5. WTC-11 (male) and SCVI-15 (male) hiPSC lines were karyotyped when (a, b) grown as adherent cultures, (a) WTC-11, (b) SCVI-15; (c, d) post-serial passaging in the

Ambr® 250 modular, (c) WTC-11, (d) SCVI-15; and (e, f) post-culture in the BioStat® B-DCU, (e) WTC-11, (f) SCVI-15. Passage number and karyotyping results are shown. 20 cells are karyotyped per sample; square brackets indicate the number of cells containing the specified karyotypic features.

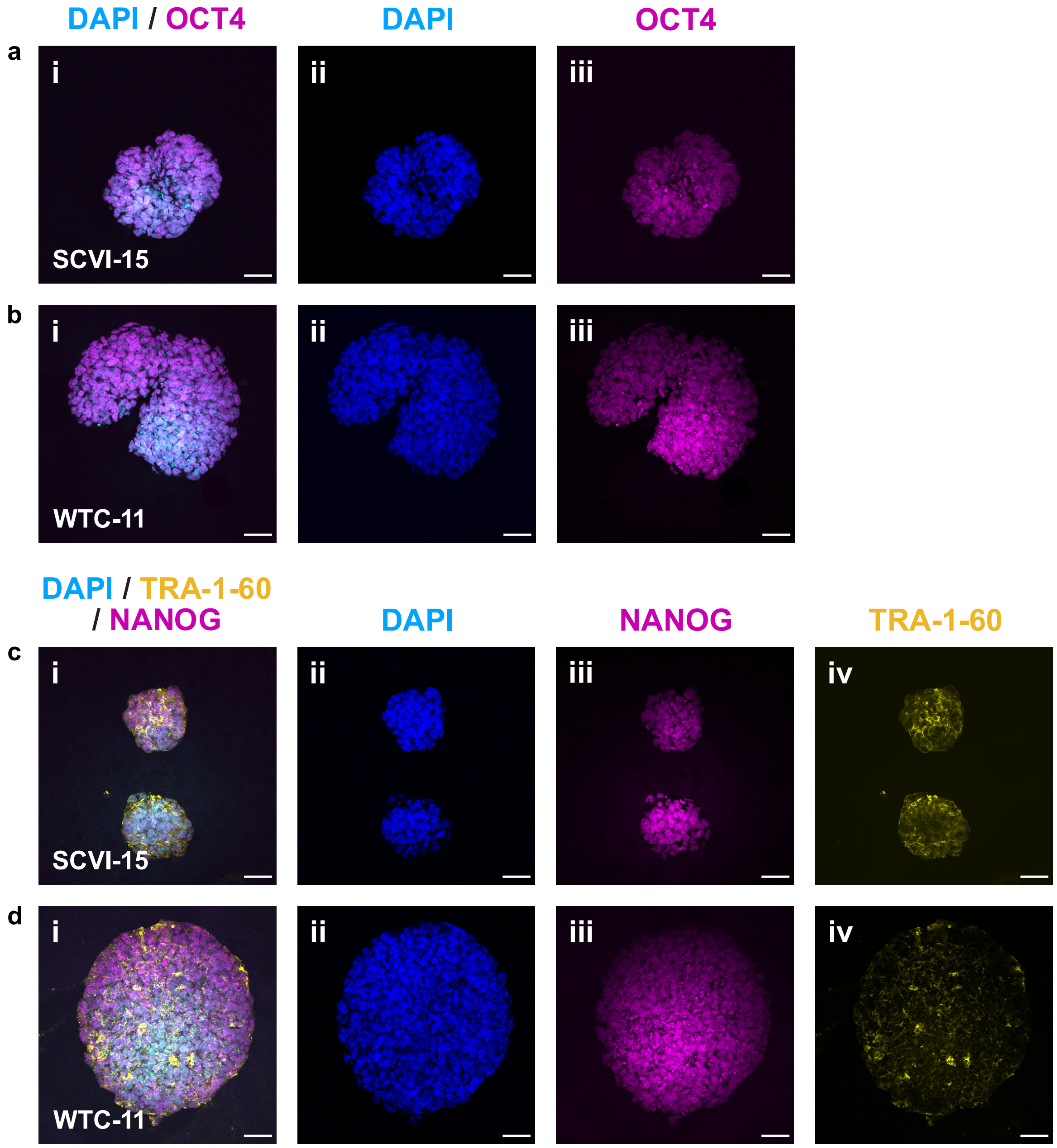

Figure S6. Immunofluorescence images of cryosectioned SCVI-15 and WTC-11 hAs on day 3 of suspension culture in the Ambr® 250 modular bioreactor. (a) SCVI-15 and (b) WTC-11 hAs stained for OCT4 and with DAPI. (i) Composite, (ii) DAPI, (iii) OCT4. (c) SCVI-15 hAs and (d) WTC-11 hAs stained for NANOG, TRA-1-60, and DAPI. (i) Composite, (ii) DAPI, (iii) NANOG, (iv) TRA-1-60. (a - d) Scale bar, 50 µm.

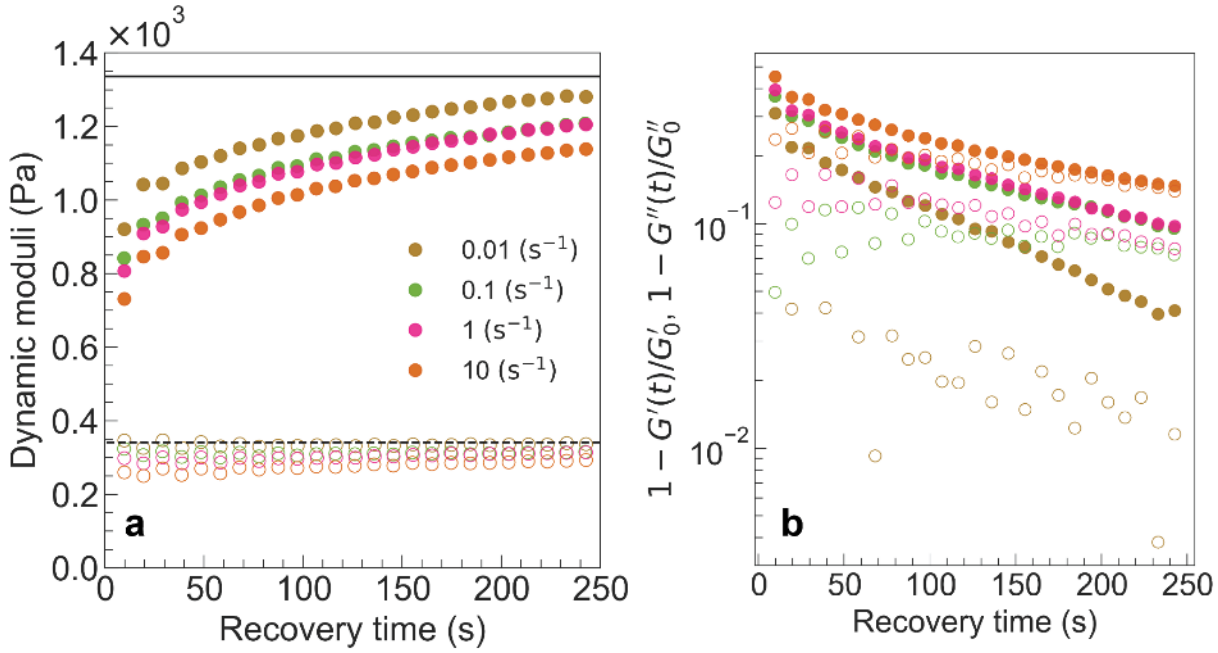

Figure S7: **Recovery of dynamical responses for SCVI-15 hA bioinks (solid circles G'; hollow circles G'') upon flow cessation at varying strain rates.** (a) $G'(t)$ and $G''(t)$ plotted against recovery time upon the cessation of each strain rate, measured at 1 rad/s at an amplitude of 1%. Solid and dashed lines are the dynamical responses at intact state under the same experimental conditions, $G_0' = 1335.9$ Pa and $G_0'' = 340.6$ Pa, extracted from independent oscillation measurements. (b) Normalized temporal evolution of the distance between $G'(t)$ and G_0' (solid markers), and $G''(t)$ and G_0'' (hollow markers) against recovery time upon the cessation of each strain rate. All measurements are performed at 20°C.

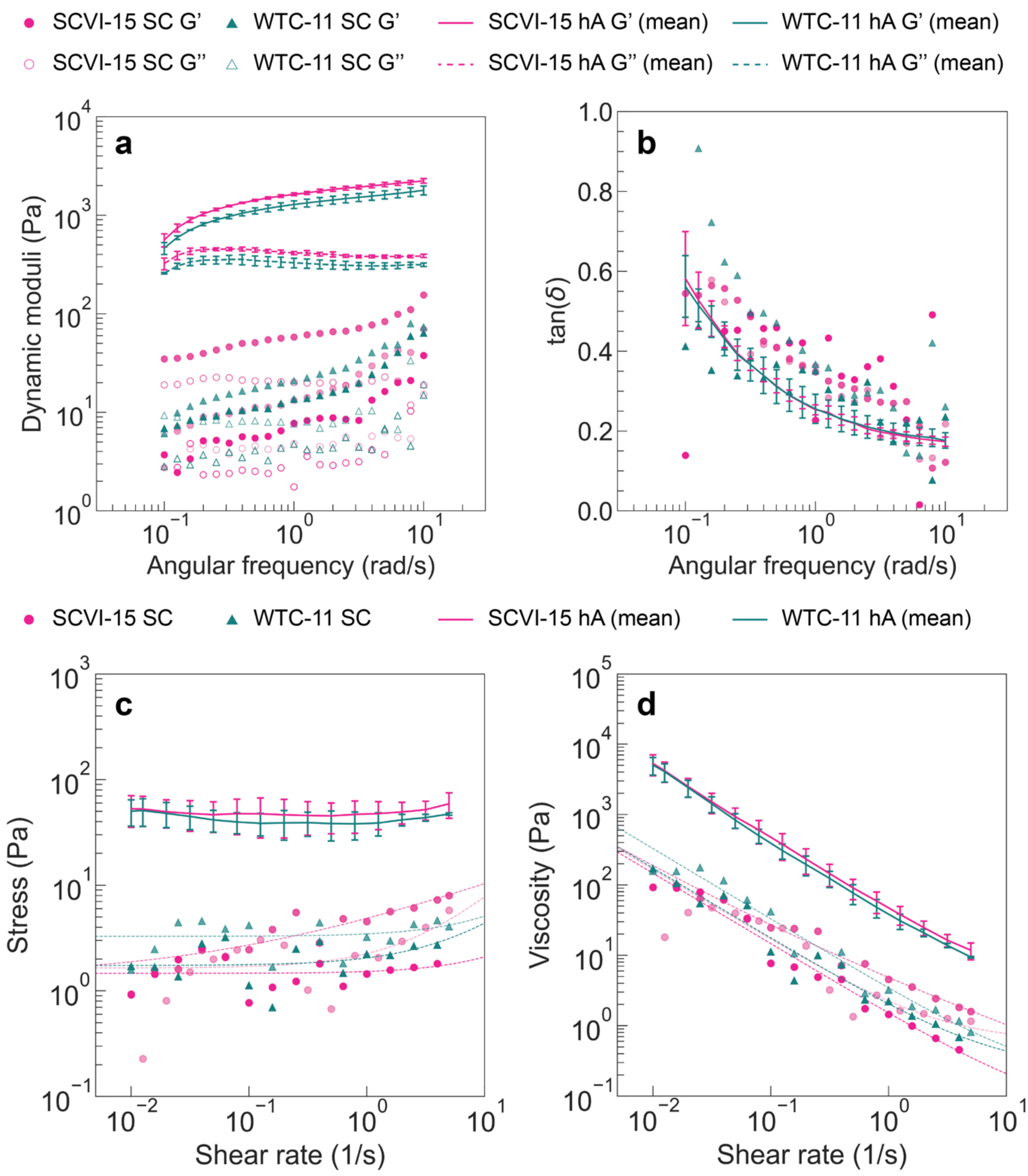

Figure S8: **Rheological characterizations of dense slurries of single SCVI-15 and WTC-11 cells**. (a) Frequency response of SCVI-15 (pink) and WTC-11 (teal) single cells (SCs) measured at 0.1 - 10 rad/s at a fixed amplitude of 1%. Filled circles and triangles are storage moduli values and empty circles and triangles are loss moduli values. Solid (storage moduli) and dashed (loss moduli) lines are the mean ± s.d. values of G' and G'' from SCVI-15 (pink) and WTC-11 (teal) hA bioinks for references. (b) Ratio of G'' and G' (tan δ) extracted from SCVI-15 SCs (pink markers) and WTC-11 SCs (teal markers). Solid lines are mean ± s.d. values of tan δ from SCVI-15 (pink) and WTC-11 (teal) hA bioinks for references. (c, d)

Steady shear stress (c) and viscosity (d) corrected for parallel-plate geometry plotted against shear rates (0.01 - 5 s^{-1}) for SCVI-15 (pink circles) and WTC-11 (teal triangles) hAs. Solid lines are mean ± s.d. value counterparts from SCVI-15 (pink) and WTC-11 (teal) hA bioinks. Dashed lines are fitting lines from the modified Herschel-Bulkley model (Equation 1). SCVI-15 hAs and SCs, n = 3; WTC-11 hAs and SCs, n = 2. All measurements are performed at 20°C.

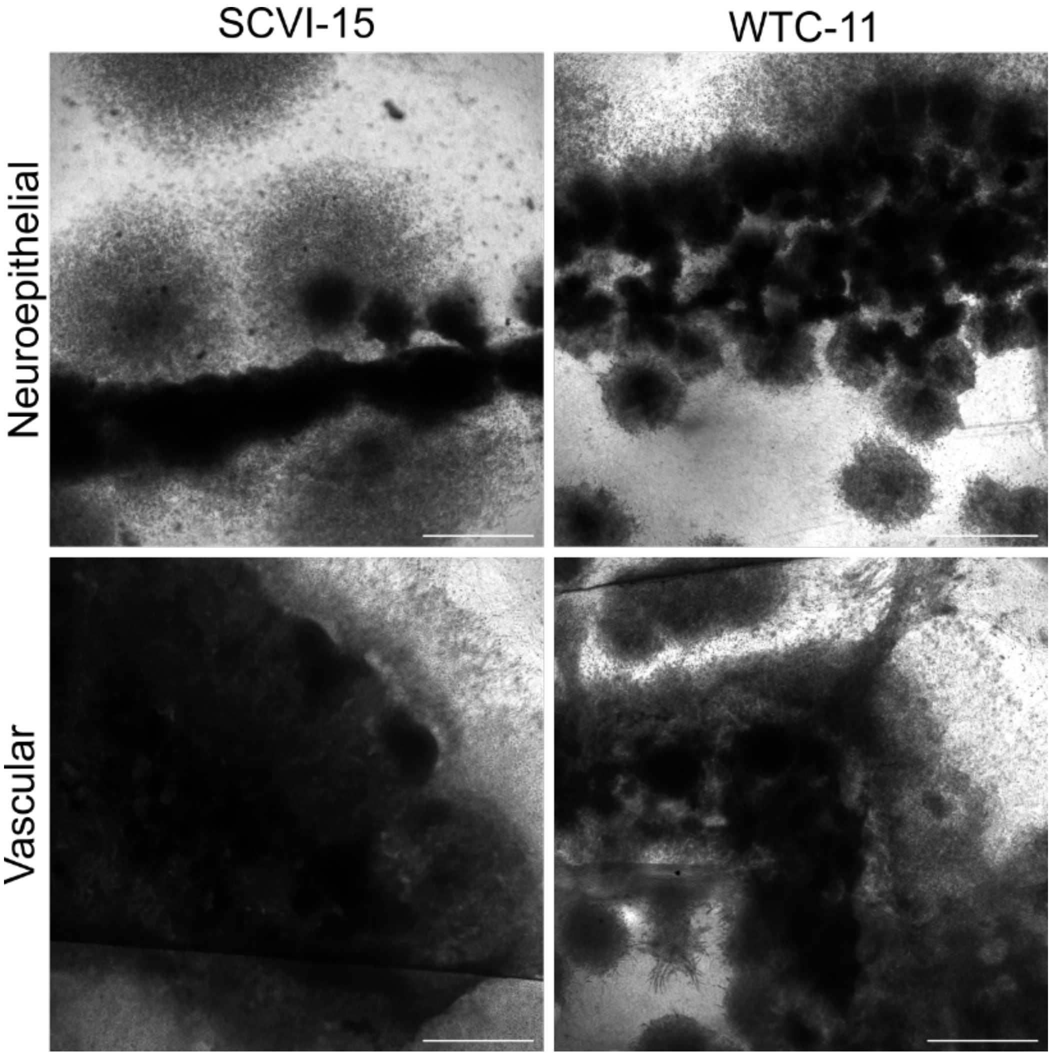

Figure S9. Brightfield images of hAs after 10 days after bioprinting and differentiation into neuroepithelium or vascular cells. Scale bars, 1000 μm.

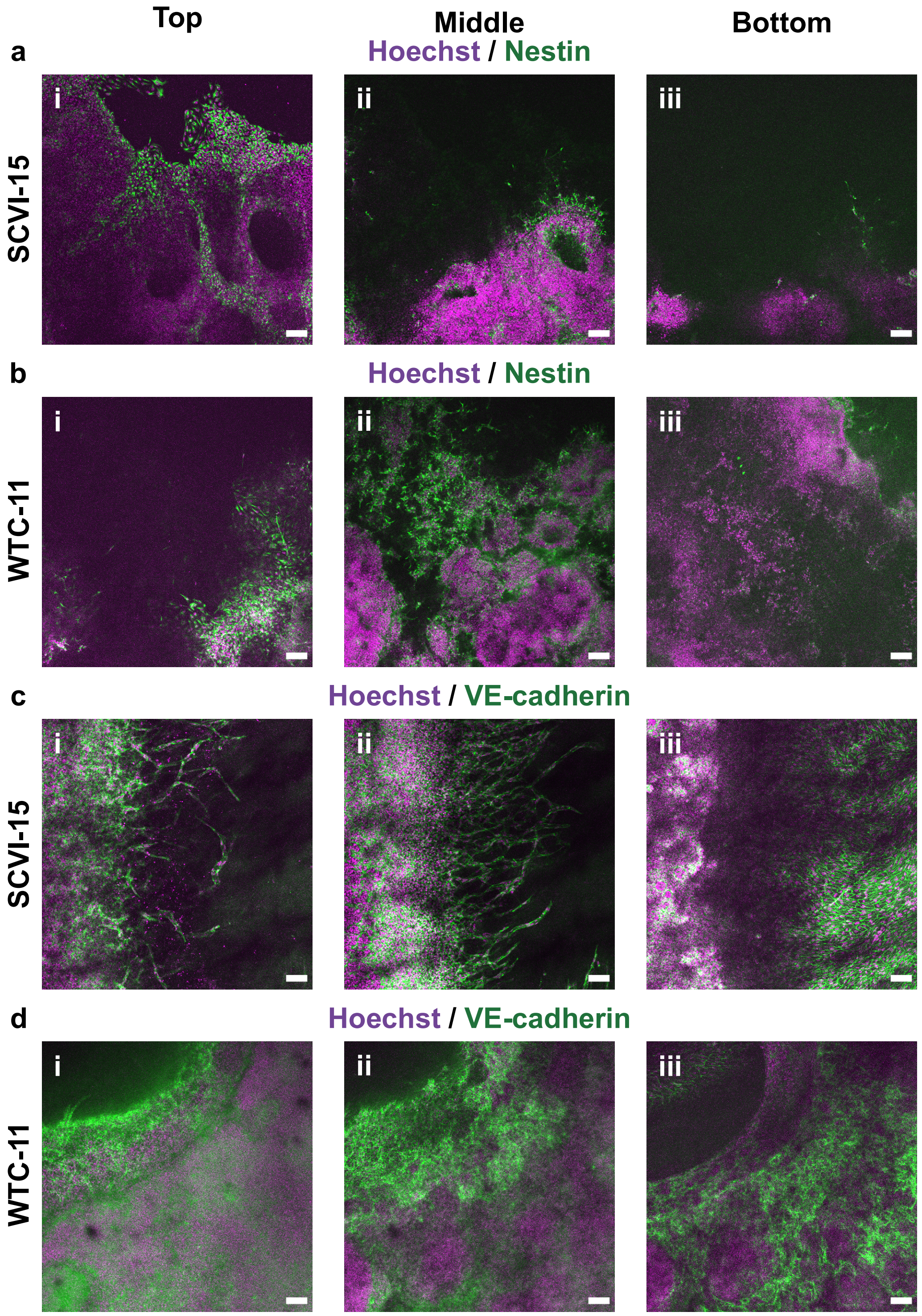

Figure S10. Differentiation throughout the depth of bioprinted tissues. (a, b) Single z-slice confocal images of day 10 neuroepithelium SCVI-15 (a) and WTC-11 (b) tissues immunostained for Nestin (green) and Hoechst (magenta) at the top (i), middle (ii), and bottom (iii, closest to coverslip). (c, d) Single z-slice confocal images of day 10 vascular SCVI-15 (c) and WTC-11 (d) tissues immunostained for VE-cadherin (green) and Hoechst (magenta) at the top (i), middle (ii), and bottom (iii, closest to coverslip). (c) SCVI-15 and (d) WTC-11 day 10 vascular squares are shown. Scale bars, 100 µm.

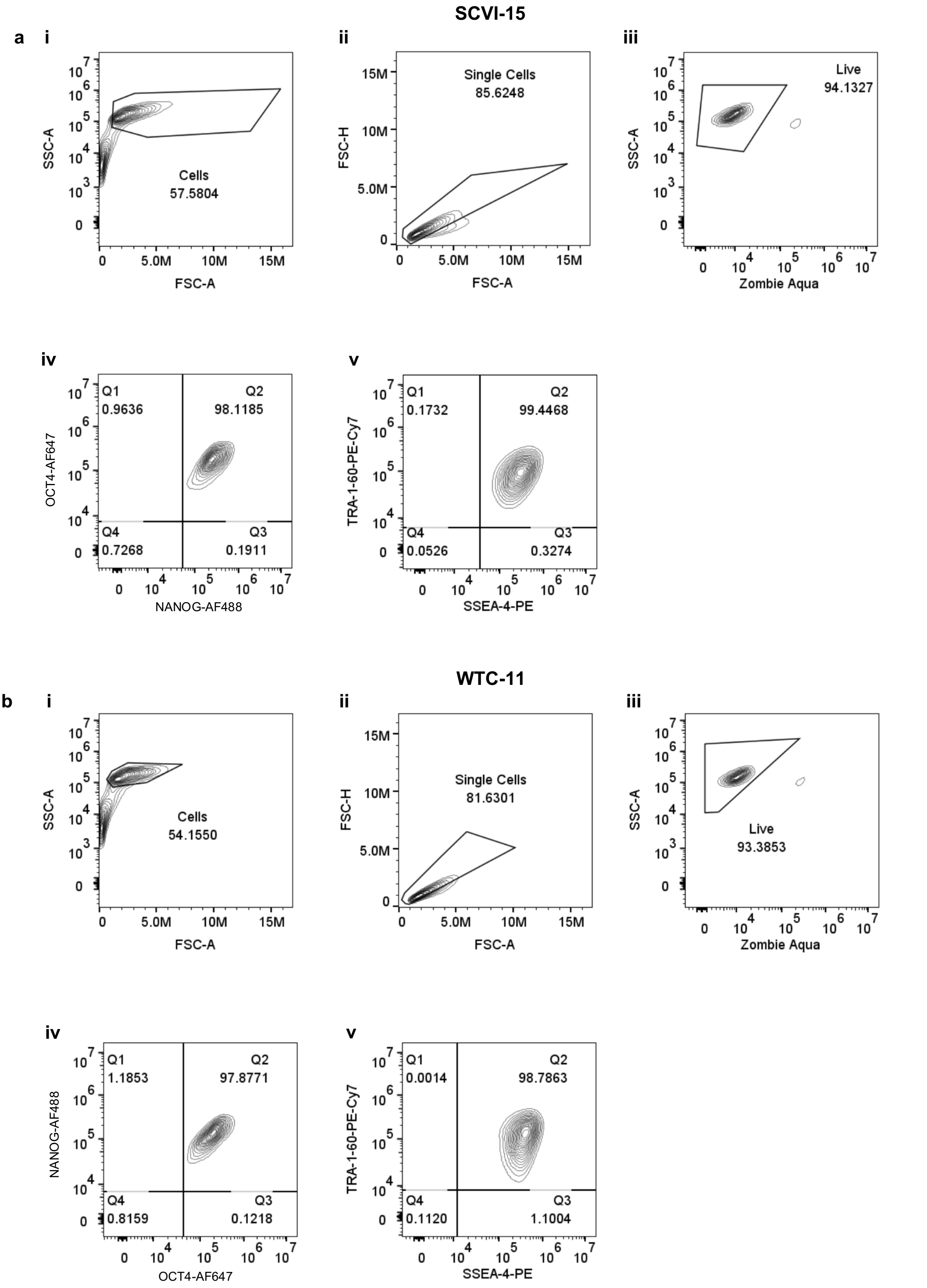

Figure S11. Example gating strategy for flow cytometry analysis of OCT4+ NANOG+ TRA-1-60+ SSEA-4+ cell populations in (a) SCVI-15 and (b) WTC-11 day 3 hAs. Examples are from the third passage SCVI-15 hAs in the serial passage experiments. Each plot is generated in hierarchical order in the following sequence: (i) cells were gated using side scatter (SSC-A) and forward scatter (FSC-A), (ii) doublets were excluded, (iii) dead cells were excluded, (iv)

NANOG+ OCT4+ cells were included, (v) NANOG+ OCT4+ SSEA-4+ TRA-1-60+ cells were included.

Table S1. Factor settings in the series of conditions tested in the DoE (design of experiments). The factors explored were initial speed, final speed, and seeding density.

Experiment Name	Initial Speed [RPM]	Final Speed [RPM]	Seed Density [k cells/mL]	PVA [mg/mL]
S_a	150	250	190	0
S_b	200	250	187	0
S_{b2}	200	250	166	0
S_{low}	100	250	117.5	4
S_c	150	250	191.5	4
S_d	200	300	89.76	0
S_e	200	300	155	0
S_f	200	400	153.6	0
S_{f2}	200	400	110.04	0
S_g	200	400	217.5	0
S_h	300	400	119.5	0
S_{high}	200	500	204.12	0
S_{mid}	200	200	214.72	0
S_i	200	275	205.92	0
S_j	300	300	187	0

Table S2. Required responses for predicting optimal culture conditions for desired pluripotency marker expression, circularity and aggregate sizes.

Name	Abbreviation	Units	Transform	Condition	Objective	Min
Cell Density day 5	CD5	M/mL	None	Required	Maximize	1
SSEA-4 day 5	SEA45	%	NegLog: -10Log(100-Y)	Required	Maximize	90
TRA-1-60 day 5	TRA5	%	NegLog: -10Log(100-Y)	Required	Maximize	90
NANOG day 5	Nan5	%	None	Required	Maximize	90

Fold Expansion day 5	FE5		Log: 10Log(Y)	Required	Maximize	10
Circularity day 5	Cir5		None	Required	Maximize	0.8
hA day 5 250 - 300 Cubic	E523C	%	None	Required	Maximize	5
hA day 5 300 - 450 Cubic	E534C	%	None	Required	Maximize	40

Table S3. Observed responses based on the prediction model set-up using parameters described in Table S2.

Name	Abbreviation	Units	Transform	Condition	Objective	Min
Cell Density day 3	CD3	M/mL	None	Observed	Predicted	1
hA day 3 250 - 300	hA323	%	None	Observed	Predicted	5
hA day 3 300 - 450	hA334	%	Log: 10Log(Y)	Observed	Predicted	2
hA day 5 250 - 300	hA523	%	None	Observed	Predicted	20
hA day 5 300 - 450	hA534	%	None	Observed	Predicted	12
SSEA-4 day 3	SEA43	%	NegLog: -10Log(100-Y)	Observed	Predicted	90
TRA-1-60 day 3	TRA3	%	NegLog: -10Log(100-Y)	Observed	Predicted	90
NANOG day 3	Nan3	%	NegLog: -10Log(100-Y)	Observed	Predicted	90
Fold Expansion day 3	FE3		None	Observed	Predicted	15
Circularity day 3	Cir3		None	Observed	Predicted	0.8
Diameter day 3	Dia3		Log: 10Log(Y)	Observed	Predicted	
Diameter day 5	Dia5		Log: 10Log(Y)	Observed	Predicted	
hA day 3 250 - 300 Cubic	E323C	%	None	Observed	Predicted	10
hA day 3 300 - 450 Cubic	E334C	%	Log: 10Log(Y)	Observed	Predicted	35

Table S4. Antibodies used in flow cytometry analysis.

Antibody	Manufacturer	Catalog No.	Dilution	RRID
Zombie Aqua	BioLegend	423101	1:1000	N/A
PE anti-human SSEA-4 Antibody	BioLegend	330406	1:200	AB_1089206
PE Mouse IgG3 κ Isotype Ctrl Antibody	BioLegend	401320	1:1600	AB_10683168
PE/Cyanine7 anti-human TRA-1-60-R Antibody	BioLegend	330620	1:200	AB_2728286

PE/Cyanine7 Mouse IgM, κ Isotype Ctrl Antibody	BioLegend	401627	1:400	N/A
OCT3/4 Mouse, Alexa Fluor 647	BD Pharmingen	560329	1:20	AB_1645318
anti-IgG1 κ, Mouse Alexa Fluor™ 647	BD Pharmingen	557714	1:320	AB_396823
NANOG Mouse anti-Human Alexa Fluor 488	BD Pharmingen	560791	1:20	AB_1937305
anti-IgG1 κ, Mouse Alexa Fluor™ 488	BD Pharmingen	557702	1:80	AB_396811
SOX17 Mouse anti Human, Alexa Fluor 647	BD Pharmingen	562594	1:5	AB_2737670
anti-IgG1 κ, Mouse , Alexa Fluor™ 647	BD Pharmingen	557714	1:83.3	AB_396823
Nestin Mouse Anti-Human Alexa Fluor® 647	BD Pharmingen	560393	1:5	AB_1645170
anti-IgG1 κ, Mouse Alexa Fluor™ 647	BD Pharmingen	557714	1:10	AB_396823
CD144 Mouse Anti-Human, PerCP-Cy™5.5	BD Pharmingen	561566	1:20	AB_10715835
anti-IgG1 κ Mouse PerCP-Cy™5.5	BD Pharmingen	550795	1:40	AB_393885
Cardiac Troponin T, Mouse Anti-Human, Alexa Fluor® 647	BD Pharmingen	565744	1:40	AB_2739341
anti-IgG1 κ, Mouse, Alexa Fluor™ 647	BD Pharmingen	557714	1:20	AB_396823

Table S5. Antibodies used in immunofluorescence

Antibody	Manufacturer	Catalog No.	Dilution or concentration	RRID
NANOG	Cell Signaling	4893S	1:200	AB_10548762
OCT4	Abcam	ab181557	1:200	AB_2687916
SSEA	BioLegend	330406	1:200	AB_1089206
TRA-1-60	BioLegend	330606	1:100	AB_1227812
Cardiac Troponin T	Abcam	ab8295	1:200	AB_306445
NKX2.5	Cell Signaling Technologies	8792S	1:400	AB_2797667
Sarcomeric α-actinin	Abcam	ab137346	1:200	N/A
VE-cadherin	Cell Signaling Technologies	2500S	1:400	AB_10839118
Calponin 1	Abcam	ab46794	1:200	AB_2291941

CD31	Abcam	ab9498	1:200	AB_307284
E-cadherin	R&D Systems	MAB1838-100	1:200	AB_2076806
CDX2	Fisher Scientific	RM2116S0	1:100	N/A
SOX17	R&D Systems	AF1924	1:100	AB_355060
TUJ1	R&D Systems	MAB1195	0.005 µg/µL	AB_3575
PAX6	Biolegend	901301	1:200	AB_2565003
MAP2	Sigma-Aldrich	MAB3418	0.005 µg/µL	AB_94856
N-cadherin	Cell Signaling Technologies	14215S	1:200	AB_2798427
NEUN	Cell Signaling Technologies	24307T	1:200	AB_2651140
Nestin	Abcam	ab6320	1:200	AB_308832
SOX2	R&D Systems	AF2018	0.01 µg/µL	AB_355110
Secondary antibodies and reagents				
Alexa Fluor 488 Donkey anti-mouse	Life Technologies	A11055	1:400	AB_2534102
Alexa Fluor 555 Goat anti-rabbit	Life Technologies	A21428	1:400	AB_141784
Alexa Fluor 647 Donkey anti-rabbit	Life Technologies	A31573	1:400	AB_2536183
Alexa Fluor 647 Goat anti-mouse	Life Technologies	A21235	1:400	AB_2535804
Alexa Fluor 488 Phalloidin	Invitrogen	A12379	1:200	N/A
DAPI	Millipore Sigma	D9542	300 nM	N/A
Hoechst 33342	Apex Bio	A3472	1:1000	N/A

Supplemental Methods: Modified Herschel-Bulkley model with the addition of thixotropic contributions

The non-monotonic trend in the stress curve in Figure 4d can be modeled as a result of the counteracting effects of shear-induced structural destruction and spontaneous re-establishment of cell- or hA-spanning structures. This temporal reconstruction is described as thixotropic responses, which can be added to the original Herschel-Bulkley model to better describe the complex flow behavior of the studied bioinks.

We noticed from **Figure S7b** that the recovery of dynamical responses at varying strain rates (from 0.01 s^{-1} to 10 s^{-1}) can be described by an exponential dynamic governed by a broadly constant timescale. Based on this observation, we approximate the structural evolution by a simple first-order differential equation [1] as

$\dot{\Theta} = -\Theta|\dot{\gamma}| + (1 - \Theta)/\lambda$. (Equation S1)

Here, the first and second terms on the right-hand side refer to the rate of shear-induced destruction and spontaneous reconstruction, respectively. The integrity of the hA-spanning network can be quantified by a dimensionless structural parameter Θ, where $\Theta = 0$ corresponds to complete destruction and $\Theta = 1$ corresponds to a fully recovered network.

Furthermore, the yield stress can be expressed by this structural parameter [2] as

$\sigma_y(\Theta) = \sigma_0\Theta$, (Equation S2)

which leads to a modified Herschel-Bulkley model with the addition of thixotropic behavior by replacing the yield stress with σ_y as

$\sigma(\dot{\gamma}) = \sigma_y(\Theta) + K\dot{\gamma}^n$, (Equation S3)

The steady-state stress can thus be calculated from Equation S1 and S3, given that $\dot{\Theta} = 0$ as

$\sigma(\dot{\gamma}) = \sigma_0/(1 + \lambda\dot{\gamma}) + K\dot{\gamma}^n$, (Equation S4)

where the first term corresponds to an apparent yield stress that varies with strain rates. In contrast to the original Herschel-Bulkley model, Equation S4 leads to a non-monotonic trend of the stress curve against strain rates.

In addition, upon the cessation of a steady-state flow, the recovery of the structural parameter can be calculated by letting $\dot{\gamma} = 0$ in Equation S1 as

$\Theta(t) = 1 - (1 - \Theta_0)\exp(-t/\lambda)$, (Equation S5)

where Θ_0 is the initial structural parameter upon the flow cessation ($t = 0$).

In general, the yield stress can be connected to the plateau storage modulus ($G_0 = \lim_{\omega \to 0} G'$) by $\sigma_y = \sigma_0 \Theta = G_0 \gamma_c$ [3], where γ_c is the critical strain beyond which non-linear dynamical responses arise. As a result, the recovery of dynamic moduli follows a similar trend as the structural parameter, which is predicted to exponentially approach the static asymptote with the timescale independent of the imposed shear rate. This prediction agrees well with **Figure S7b**.

With the integration of thixotropic behavior, the proposed modified Herschel-Bulkley model captures both the steady-state and transient flow responses. Such thixotropic behavior can be further connected to the self-healing feature of a bioink, which is critical to design and optimize the high-speed bioprinting strategy, if good shape fidelity is desired.

Movie S1. VE-cadherin+ lumen-like structures in SCVI-15 and WTC-11 cells differentiated into endothelial cells. Video shows two juxtaposed fields of view per cell line. Each frame is spaced 5 µm apart. Playback speed: 2 fps. Scale bar: 20 µm.

Movie S2. Spontaneous contraction of day 10 WTC-11 cardiac organoids. Scale bar: 100 µm.

Movie S3. Bioprinting of hA bioink into collagen-Matrigel matrix.

References

[1] A. Mujumdar, A. N. Beris, A. B. Metzner, *J Non-newton Fluid* 2002, *102*, 157.
[2] C. J. Dimitriou, G. H. McKinley, *Soft Matter* 2014, *10*, 6619.
[3] M. Dinkgreve, J. Paredes, M. M. Denn, D. Bonn, *J Non-newton Fluid* 2016, *238*, 233.
